# A protocol for a three-arm cluster randomized controlled superiority trial investigating the effects of two pedagogical methodologies in Swedish preschool settings on language and communication, executive functions, auditive selective attention, socioemotional skills and early maths skills

**DOI:** 10.1186/s40359-018-0239-y

**Published:** 2018-06-19

**Authors:** Tove Gerholm, Thomas Hörberg, Signe Tonér, Petter Kallioinen, Sofia Frankenberg, Susanne Kjällander, Anna Palmer, Hillevi Lenz Taguchi

**Affiliations:** 1 Department of Linguistics, Stockholm University, Stockholm, Sweden; 2 Department of Psychology, Stockholm University, Stockholm, Sweden; 3 Department of Child and Youth Studies, Stockholm University, Stockholm, Sweden

**Keywords:** Intervention, Executive functions, Selective attention, Language skills, Early maths skills, Communication skills, Socioemotional skills, Group-based learning, Digital learning

## Abstract

**Background:**

During the preschool years, children develop abilities and skills in areas crucial for later success in life. These abilities include language, executive functions, attention, and socioemotional skills. The pedagogical methods used in preschools hold the potential to enhance these abilities, but our knowledge of which pedagogical practices aid which abilities, and for which children, is limited. The aim of this paper is to describe an intervention study designed to evaluate and compare two pedagogical methodologies in terms of their effect on the above-mentioned skills in Swedish preschool children.

**Method:**

The study is a randomized control trial (RCT) where two pedagogical methodologies were tested to evaluate how they enhanced children’s language, executive functions and attention, socioemotional skills, and early maths skills during an intensive 6-week intervention. Eighteen preschools including 28 units and 432 children were enrolled in a municipality close to Stockholm, Sweden. The children were between 4;0 and 6;0 years old and each preschool unit was randomly assigned to either of the interventions or to the control group. Background information on all children was collected via questionnaires completed by parents and preschools. Pre- and post-intervention testing consisted of a test battery including tests on language, executive functions, selective auditive attention, socioemotional skills and early maths skills. The interventions consisted of 6 weeks of intensive practice of either a socioemotional and material learning paradigm (SEMLA), for which group-based activities and interactional structures were the main focus, or an individual, digitally implemented attention and math training paradigm, which also included a set of self-regulation practices (DIL). All preschools were evaluated with the ECERS-3.

**Discussion:**

If this intervention study shows evidence of a difference between group-based learning paradigms and individual training of specific skills in terms of enhancing children’s abilities in fundamental areas like language, executive functions and attention, socioemotional skills and early math, this will have big impact on the preschool agenda in the future. The potential for different pedagogical methodologies to have different impacts on children of different ages and with different backgrounds invites a wider discussion within the field of how to develop a preschool curriculum suited for all children.

**Electronic supplementary material:**

The online version of this article (10.1186/s40359-018-0239-y) contains supplementary material, which is available to authorized users.

## Background

In Sweden (2016), 84% of all children aged 1–5 years old, and 95% of children aged 4–5 years old, spend between 15 and 50 h per week in preschools [1]. This makes preschool an immensely influential learning ground. The Swedish curriculum focuses heavily on fostering democratic citizens and thus on a vast array of primarily socioemotional skills. More specific learning skills within language development and STEM (Science, Technology, Engineering and Mathematics) areas have only more recently been emphasized as curriculum goals to strive for, but with no specific aim to achieve and no consistent methods of implementing these aims have yet been set forth [2]. How this learning should be undertaken is not specified and different preschools chose different teaching practices [3]. Two pedagogical methods which are frequently used, albeit in different proportions and in different forms, are socioemotional learning [3] and learning through digital material [4]. Still, our evidence-based knowledge of the effectiveness of these and other pedagogical practices is low or non-existent. To our knowledge, only two controlled studies have been executed in the Swedish preschool context investigating cognitive training with digital materials: one with 4–5-year-old children [5] and one in the so-called preschool-class, which follows regular preschool and precedes compulsory schooling [6]. No controlled study has been undertaken regarding the effects of socioemotional learning.

During the preschool years, children move from developing prelinguistic skills to becoming full-blown language users. This period is also characterized by the development of executive functions and selective attention, and by the acquisition and practice of culturally coded behaviours such as socioemotional regulation and interaction skills [7, 8]. It is well established that the acquisition and development of these skills is to some extent guided by the child’s background in terms of socioeconomic status (SES). Parental SES continues to influence the child’s developmental curve after preschool and is highly correlated to later school achievements and career opportunities [9, 10].

By acquiring the ambient language(s), a child gains access to society at large. It is through verbal and nonverbal interactions with peers and adults that an individual creates a social life for him or herself and learns new skills. Having a rich vocabulary and the ability to express oneself narratively in oral as well as in written language gives a child access to parts of life that are central to future choices and opportunities. It is well documented that children from different socioeconomic backgrounds reach different outcomes as to vocabulary size and narrative skills [9, 11]. This is thought to relate to the different home environment these children have, where a higher socioeconomic status appears to correspond to parents who spend more time talking to and with their children, who use a richer and more nuanced vocabulary, and who encourage their child to explore and use a rich language of their own [12, 13].

In parallel to and at times closely intertwined with language, cognitive abilities develop rapidly during the child’s first years [14, 15]. Among these abilities are executive functions (EF), top-down mental processes generally considered to consist of three core components: working memory, inhibition (including selective/focused attention) and cognitive flexibility/shifting [16–18]. These executive function components are used to organize higher-order control of thinking and behaviour [8] and serve as the foundation for higher cognitive functions such as decision-making, planning and problem solving [8, 18].

The ability to control one’s attention is a crucial component of learning [8, 19]. There have been many different functional categorisations of attention in the literature. Imaging data have, however, supported the presence of three networks related to different aspects of attention that carry out the functions of alerting, orienting and executive attention [20]. Alerting is defined as achieving and maintaining a state of sensitivity to incoming information; orienting means to selectively attend to something and to ignore what is irrelevant; and executive attention monitors and resolves conflict among thoughts and feelings and is involved in planning/decision-making [21]. Executive attention seems to be most important for future academic success, although it is intertwined with and will involve either the alert state or executive control networks, depending on whether the information is sensory or comes from prior memories, etc. [16, 21, 22]. Very small children have all of these capacities, but speed and efficiency increase with age and by means of practice and conscious training [23]. As with executive functions and language, children growing up in low socioeconomic circumstances are at risk for not developing to their best potential in these skills [24].

EF and language skills together make up children’s ability to handle socioemotional aspects of life. This includes being able to regulate emotional experiences and engage in positive and constructive interactions with peers and adults [8, 25]. Socioemotional skills also determine our ability to interact with others, both peers and adults, and to do this in a flexible and considerate manner [26, 27]. Interactional skills of this kind come about through socialization processes where children learn through interaction with more skilled models, such as older siblings and parents [28–30].

Like language, executive functions and attention have been positively correlated to a number of skills and outcomes related to wellbeing such as social and academic success [31, 32], and specific skills such as mathematics [22]. Executive functions are also a good predictor for maths skills development [33].

It is well documented that a good command of language, EF, attention, and socioemotional skills correlate positively with later success in school and work, and have an immense impact on the child’s socioemotional life and interactions with peers and adults [31, 32]. A growing body of research supports that the abilities children acquire during the preschool years, and which are highlighted in the preschool curriculum, are malleable, develop in relation to context, and can be trained [22, 34]. On the whole, the preschool setting holds the potential for enhancing children’s development in areas central to their future life prospects. The learning ground of the preschool could, in particular, aid children who are at a disadvantage in terms of background support in the form of engaged social networks that are also well integrated into society. However, it is not clear how particular skills (like language, executive functions, attention and maths) are to be taught and practiced. Based on the introduced body of research and our knowledge of already present pedagogical methodologies in the Swedish preschool settings, two interventions were designed to test the possibility of enhancing children’s abilities in the areas of language, EF, selective attention, socioemotional skills and early maths skills.

Social-emotional learning (SEL) practices have been suggested as strategies to foster children’s executive functions and attention skills, social awareness, relationship skills and responsible decision-making [35, 36]. These competencies, in turn, are expected to provide a foundation for better adjustment and academic performance as reflected in more positive social behaviours, fewer conduct problems, less emotional distress and improved test scores and grades [8, 37, 38]. In the present project, SEL was developed into Social and Emotional Material Learning (SEMLA) in order to strengthen children’s interactional, language-dependent capabilities and highlight the potential use of multimodal learning through materials as well as interactional practices [39]. SEMLA is a group-based intervention where the participating children, in groups of 6–8, are guided by trained preschool staff working on a specific explorative project, in this case “How to live and get around 100 years from now”. SEMLA aims to enhance the child’s attention, executive functions, language, socioemotional skills and early maths skills by way of introducing a creative construction project in a material space filled with inspirational materials for the children to engage with and sensitive to their individual curiosity, motivation, and desires.

The SEMLA intervention was contrasted with a digital learning intervention, DIL. Digital Individual Learning (DIL) for Body and Mind aims to enhance the child’s executive functions by way of brain training and attention-enhancing exercises in combination with training early maths and number sense [22]. Different programs have been developed in order to train attention and executive functions, with the aim of having these important abilities transfer to other areas and contexts of use [34]. Some programs use digital devices to train specific skills such as working memory, while others promote different types of exercises such as training mindfulness [40–45]. DIL is an individual design in which a time-limited, structured pedagogical intervention is implemented through repeated interaction with learning materials such as an early math software The Magical Garden,^1^ which is an interactive game with exercises that progressively advance in difficulty and are scaffolded by teachers [46]. The exercises for body and mind were inspired by the Brain Development Lab [47]. The aim is to create a lasting and transferable effect in bodily function (including neurological) that will improve the child’s ability to understand and control his/her body and mind. As the theme of the game is mathematics and number sense, an additional aim of the intervention is to enhance the child’s early maths skills.

As an active control, the study used BRUK, a self-evaluative tool administered by the Swedish National Agency for Education [2]. It is developed as a support for pedagogical staff and includes questions and guidelines on systematic quality work in areas relating to working methods, goals and goal fulfillment. The tool is designed for and used by the pedagogical staff themselves.

## The current study

This paper describes the design and implementation of an intervention RCT study, where two contrasting pedagogical practices are evaluated in terms of their ability to enhance preschool children’s language, executive functions, attention, socioemotional skills and early maths skills during a 6-week intensive pedagogical practice period. Prior to analysing the data, the procedures chosen are rigorously described in order to facilitate replication and obstruct perils inherent to result fishing. This study protocol adheres to the guidelines of the SPIRIT checklist of protocol items [48].

The project addresses the following research questions:

### Research questions

1. What are the effects of the pedagogical interventions SEMLA and DIL on selective attention and executive functions, as well as on language, communication, socioemotional and early maths skills?
2. How do any observed intervention effects on selective attention and executive functions, as well as on language, communication, socioemotional and early maths skills, differ between the SEMLA and DIL interventions?
3. To what extent are any observed effects of the SEMLA and DIL interventions mediated by executive functions, selective attention and/or language?
4. To what extent are any observed effects of the SEMLA and DIL interventions moderated by the background variables (age, sex, preschool start, preschool time, second language, medical conditions)?
5. To what extent are the background variables related to the outcome variables?
6. To what extent are the outcome variables related to each other?
7. Do any observed effects of the SEMLA and DIL interventions differ in terms of strength and variation?

### Hypotheses

On the basis of findings in earlier studies, we formulated seven sets of hypotheses corresponding to each of the seven research questions. The study aims to address the research questions by testing each of these hypotheses, listed in Table 1 below. For each hypothesis, Table 1 shows the outcome variable that is hypothesized to be affected, the intervention(s) or predictor either to affect or to be correlated with that outcome variable, the kind of effect or relationship that the hypothesis involves, for research questions 3 and 4, the mediating or moderating variables, the measure(s) used to estimate the outcome variable of the hypothesis at hand, and, finally, the kind of analysis method that will be conducted in order to test the hypothesis. In the following sections, we describe the method, the study design, the measurements and the analyses that will be used to test these hypotheses.

**Table 1:** Hypotheses corresponding to each of the seven research questions described in terms of the affected outcome variable, the affecting intervention(s) or correlated predictor, the effect or relationship type, the mediating or moderating variable (if applicable), and analysis method used for hypothesis testing

Research question	Outcome variable(s)	Intervention/ Predictor	Effect	Mediator / moderator	Measure(s)	Analysis
RQ1: Intervention effects	EF	SEMLA & DIL	Positive intervention effects	–	Selective attention difference, EF indice difference	Mixed effects modelling
	Early Maths skills	SEMLA & DIL	Positive intervention effects	–	Maths difference	Mixed effects modelling
	Socioemotional skills	SEMLA & DIL	Positive intervention effects	–	TEC difference	Mixed effects modelling
	Communication	SEMLA & DIL	Positive intervention effects	–	Communication indice difference	Mixed effects modelling
	Language	SEMLA & DIL	Positive intervention effects	–	Language indice difference	Mixed effects modelling
RQ2: Intervention differences	Maths	SEMLA vs. DIL	Stronger effect of DIL	–	Maths difference	Planned comparisons
	EF	SEMLA vs. DIL	Differential effect	–	Selective attention difference, EF indice difference	Planned comparisons
	Communication	SEMLA vs. DIL	Stronger effect of SEMLA	–	Communication indice difference	Planned comparisons
	Language	SEMLA vs. DIL	Stronger effect of SEMLA	–	Language indice difference	Planned comparisons
RQ3: Mediating effects	Socioemotional skills	SEMLA & DIL	EF mediated effect	EF	TEC difference	Test of mediation effect
	Communication	SEMLA & DIL	EF mediated effect	EF	Communication indice difference	Test of mediation effect
	Language	SEMLA & DIL	EF mediated effect	EF	Language indice difference	Test of mediation effect
	Maths	SEMLA & DIL	EF mediated effect	EF	Maths difference	Test of mediation effect
	EF	SEMLA & DIL	Language mediated effect	Language	Selective attention difference, EF indice difference	Test of mediation effect
	EF	SEMLA & DIL	Maths mediated effect	Maths	Selective attention difference, EF indice difference	Test of mediation effect
RQ4: Moderating effects	EF	SEMLA & DIL	Negative SES moderation	SES	Selective attention difference, EF indice difference	Mixed effects interaction model
	Language	SEMLA & DIL	Negative SES moderation	SES	Language indice difference	Mixed effects interaction model
	Socioemotional skills	SEMLA & DIL	Negative SES moderation	SES	TEC difference	Mixed effects interaction model
	EF	SEMLA & DIL	Negative EF moderation	EF	EF indice	Mixed effects interaction model
	EF	SEMLA	Positive ECERS moderation	ECERS	EF indice	Mixed effects interaction model
	Maths	SEMLA	Positive ECERS moderation	ECERS	Maths difference	Mixed effects interaction model
	Socioemotional skills	SEMLA	Positive ECERS moderation	ECERS	TEC difference	Mixed effects interaction model
	Communication	SEMLA	Positive ECERS moderation	ECERS	Communication indice difference	Mixed effects interaction model
	Language	SEMLA	Positive ECERS moderation	ECERS	Language indice difference	Mixed effects interaction model
RQ5: Background-outcome relationships	Selective attention	SES	Positive relationship	–	Selective attention	Correlational / mixed effects model
	EF	SES	Positive relationship	–	EF	Correlational / mixed effects model
	Language	SES	Positive relationship	–	Language	Correlational / mixed effects model
	Communication	Sex	Higher mean for girls	–	Communication indice	t-test / mixed effects model
	Socioemotional skills	Sex	Higher mean for girls	–	TEC	t-test / mixed effects model
	EF	Sex	Higer mean for girls	–	EF indice	t-test / mixed effects model
	EF	Multilingual	Higer mean for multilinguals	–	EF indice	t-test / mixed effects model
	Maths	Other L1	Higher mean for Swedish L1 children	–	Maths	t-test / mixed effects model
	Socioemotional skills	Language	Positive relationship	–	TEC	Correlational / mixed effects model
RQ6: Background relationships	Preschool time	Preschool start	Negative relationship	–	Preschool time	Correlation / mixed model
	SECDI	Age	Positive relationship	–	SECDI	Correlation / mixed model
	Preschool start	Other L1	Higher mean for Swedish L2 children	–	Preschool start	t-test / mixed-model
	Preschool time	SES	Positive relationship	–	Preschool time	Correlation / mixed model
	SES	Multilingual	Higher mean for Swedish L1 children	–	SES	t-test / mixed-model
	SECDI	SES	Positive relationship	–	SECDI	Correlation / mixed model
RQ7: Intervention effect differences	EF	SEMLA vs. DIL	Less variation in DIL	–	Selective attention difference, EF indice difference	F-test of variance equality
	Early Maths skills	SEMLA vs. DIL	Less variation in DIL	–	Maths difference	F-test of variance equality
	Socioemotional skills	SEMLA vs. DIL	Less variation in DIL	–	TEC difference	F-test of variance equality
	Communication	SEMLA vs. DIL	Less variation in DIL	–	Communication indice difference	F-test of variance equality
	Language	SEMLA vs. DIL	Less variation in DIL	–	Language indice difference	F-test of variance equality

## Methods/design

### Study design

The study is a three-arm cluster randomized controlled superiority trial whose design and dissemination adheres to the CONSORT guidelines for evaluation of randomised controlled trials [49], the CONSORT extension for cluster trials [50], and the CONSORT extension for non-pharmacological treatment interventions [51]. The study contains two intervention conditions, Social and Emotional Material Learning (SEMLA), and Digital Individual Learning (DIL) for Body and Mind, as well as an active control condition, BRUK. The design is a 3 (Intervention) × 2 (Time) parallel-group design which is both hypothesis testing and exploratory in nature. Participants were screened for eligibility and prompted to provide informed consent at an initial stage. Once informed consent had been provided, preschool clusters were randomly assigned to interventions. Pretest measurements were performed during a two-week period directly after randomisation. The intervention period ran for 6 weeks and was directly followed by a two-week period during which posttest measurements were collected. Manipulation checks were not performed during the intervention periods. However, adherence data will be taken into account in the statistical analyses. Table 2 illustrates the study procedure.

**Table 2:**
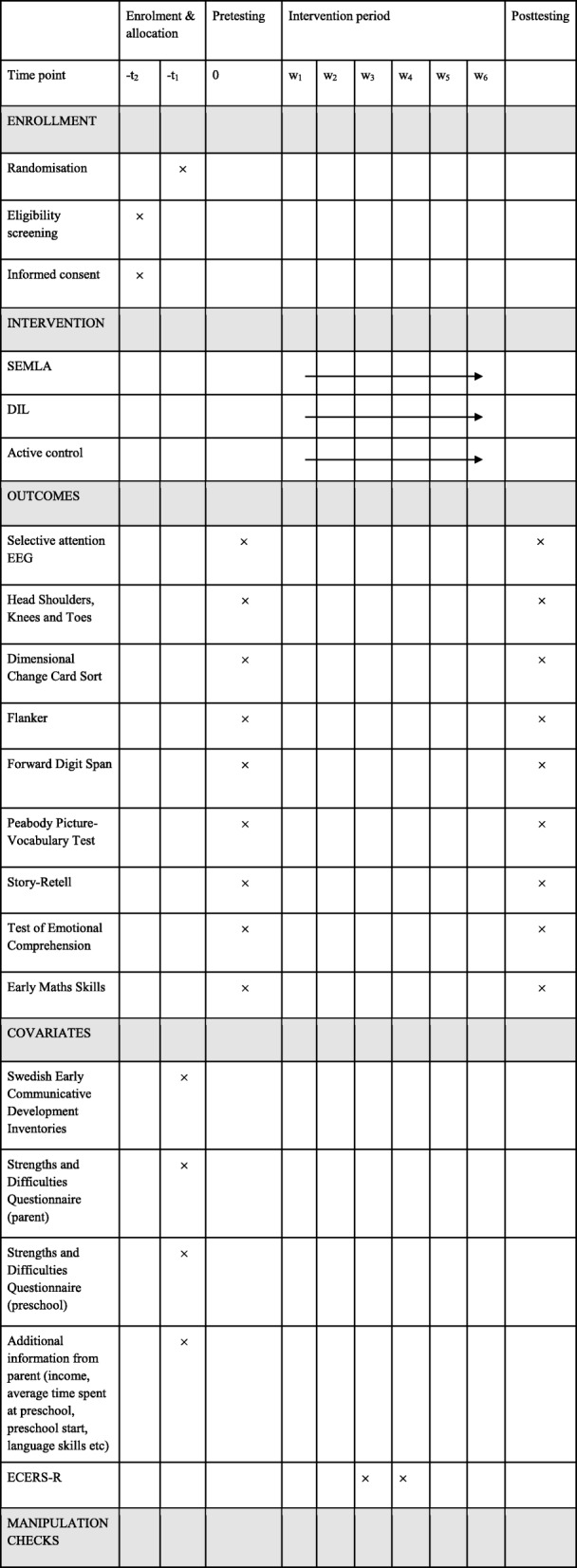
A graphical description of the procedure of the study (in accordance with the SPIRIT guidelines for study protocols)

Data collection and the implementation of the interventions was performed in three rounds, the first round ranging from the 3rd of October 2016 to the 9th of December 2016, the second from the 9th of January 2017 to the 17th of March 2017, and the third from the 20th of March 2017 to the 2nd of June 2017.

### Sample

#### Sample characteristics

The population consists of preschool children in the age range of 48 to 77 months, living in a municipality located in the eastern parts of the Stockholm metropolitan area. The sample consists of 432 children from 28 preschool units, which, in turn come from 18 preschools. Preschool units constitute clusters to which interventions are randomly assigned. Intervention assignment was constrained in the sense that units within the same preschool were always assigned either to the same intervention type or to one of the pedagogical interventions and the control group. From each preschool unit, a smaller group of children was randomly sampled (as described below) for participation in the ERP study. The size of these sub-samples was proportional to the size of the preschool unit from which they were drawn. The sample size in the ERP study is 123 individuals following attrition.

### Eligibility

All children that were 4 years or older were eligible to participate in the study. No other eligibility criteria that might have been motivated, such as a basic understanding of Swedish, were used due to ethical considerations. We didn’t want to risk individual children feeling excluded from the intervention activities during the intervention periods. All children were informed of their right to withdraw from the study at any time, and testers had to make sure participants were willing to participate at every test situation. This was done by direct questions but also through interpretation of child behaviour and follow-up questions in cases where the child appeared to be unwilling (but too shy to express this).

### Recruitment

Recruitment was performed at the level of the preschool unit. In the spring of 2016, a meeting took place with preschool managers and preschool unit staff from all preschools in the municipality. They were informed about the study and decided whether they wanted to participate on the basis of the information that was provided to them. All preschool units that expressed interest in participating were included in the study.

Children that fulfilled the eligibility criteria and provided informed consent from their caretakers were then included as participants in each cluster. Neither parents nor preschools received any payment or other incentives in order to participate, but preschool units receive follow-up feedback in terms of the ECERS-3 evaluation. The preschools were also promised continued cooperation with Stockholm University, a collaboration in which further education in the SEMLA or DIL practice would be included, along with a network (already active as the study commenced) in which researchers with findings relating to the preschool setting lectured to interested preschool staff in the municipality on a monthly basis.

### Randomisation

Each cluster was randomly assigned to one of the three conditions with equal probability. If the randomisation resulted in two or more clusters from the same preschool having different intervention assignments (i.e., SEMLA and DIL being assigned to two preschool units within the same preschool), the random assignment was performed again. Randomisation was also performed again if it resulted in obvious skewings in the age distribution across conditions. The randomization was performed in Excel by the first author. Overall, the distribution of children to the three interventions is fairly even (32, 36 and 32%).

Approximately a third of the children participated in the ERP experiment. They were selected based on a randomized priority list. If a child declined to participate or was not present, the next child on the priority list was recorded instead. In sum 139 children (64 boys, 75 girls) were recorded, while 48 children (30 boys, 18 girls) declined to participate, and 21 (13 boys, 8 girls) were not present at the preschool on the day(s) of testing. In 14 cases (3 boys, 11 girls), the priority list was ignored, such that a particularly willing child was recorded to inspire peers.

### Blinding

All preschools, units at preschools and intervention conditions were given letters for identification. The key to which preschool and unit had which letter combination was only known to the first author and the PI – who were not involved in the actual testing and intervention procedures. As an extra precaution, the data was re-named with new letter combinations prior to being delivered to the data analyst (second author).

The testers were not informed of the randomization results and do not know which preschool had which intervention. However, there is a high risk of leakage as the testers spent a lot of time at a preschool and might overhear children and staff talking, unintentionally giving away clues as to which intervention was used. It was not possible to eradicate this problem completely, but the testers were not informed as to the content of the interventions or the hypotheses of the project at large.

### Interventions

In the study, the experimental manipulation was implemented in the preschools as the three intervention conditions SEMLA, DIL and control. SEMLA and DIL are both believed to enhance executive functions, attention, and early maths skills whereas language and socioemotional skills are more clearly grounded in the SEMLA practice. The different mechanisms at work in the two pedagogical paradigms are described below. The description adheres to the guidelines of the TIDieR checklist for intervention, description and replication [52].

#### SEMLA

SEMLA aims to enhance the child’s attention, executive functions, language, socioemotional skills and early maths skills by means of introducing a creative construction project in a material space filled with inspirational materials for the children to engage with based on their individual curiosity, motivation, and desires. The following components provide the mechanisms for change:

1. Individual and group learning scaffolded by trained preschool teachers. The assumption is that SEMLA will facilitate moments of intense attention during which the child, individually or in groups, will engage with the materials provided. The small group sizes are expected to facilitate the teachers’ focused attention on each child as well as the group and will make it possible for the teachers to scaffold each child’s learning.
2. Socioemotionally supportive learning environment. Teachers in SEMLA are instructed to pay specific attention to the children’s social and emotional development during the intervention by encouraging collaboration and supporting interaction between individual children.
3. An aesthetic, playful, creative and experimental exploration is facilitated by the specific materials that are organized in a specifically assigned room, providing conditions for affectively engaging activities and focused attention. The intervention includes various types of building materials, posters of different types of buildings, and an inspirational booklet with guidelines for the preschool staff. In addition, learning tablets are used to search the Internet and for documenting the ongoing activities (pedagogical documentation). The documentation is revisited during the project in order to maintain focus, reflect on the ongoing learning process, and find inspiration for further development.

The staff was trained for four 3-h evening sessions presenting theory as well as practical exploration and creative involvement with the materials. The intention was to enhance the preschool staff’s pedagogical capacity to scaffold and emotionally support the children’s learning through a learning-by-doing intervention. Supervisors guide the teachers through an explorative project similar to the one the children would be working on in order to provide the teachers with a hands-on experience interacting with the materials.

SEMLA was implemented for 1 ½-hour sessions, 4 days a week during the 6-week intervention period. The overall focus during these weeks was to work with a project investigating “How to live and get around 100 years from now”. With support from SEMLA supervisors, staff transformed a space in the classroom for investigational practices. The space was furnished with a set of creative materials, e.g. building blocks, re-cycled materials, textiles, drawing and painting materials, tools, flashlights etc. Children were taught how to document their activities using a digital camera and an iPad and encouraged to make drawings and ‘write’/articulate instructions on the theme chosen. Teachers participated in the creations and were instructed to encourage and scaffold all children to be engaged in learning activities during the SEMLA sessions, as well as to document individual children’s learning, strengths, and difficulties in the process. The combined work of the group was used to enable further scaffolding.

#### DIL

Digital Individual Learning (DIL) for Body and Mind aims to enhance the child’s early maths skills in terms of number sense and self-regulated learning by ways of brain-training and attention enhancing exercises in combination with training early math [22].

The following components provide the mechanisms for change:

1. A package of 12 activities focusing on body awareness, breathing and attention, administered and implemented by the preschool teachers during circle time each session. In particular, two metacognitive strategies (The Bird Breath and Oh, well I can…) were introduced and trained, separately and in combination with other activities. Different materials such as posters, beanbags, balloons and pinwheels were used in the activities.
2. The digital learning game Magical Garden (MG) focusing early math and number sense administered on-line by the Education Technology Group at Lund University. The main theme of the game is for the child to solve math problems in order to collect water to use for creating a flourishing garden. By solving the problems, early maths skills are expected to improve. In addition, by applying the strategies taught in the body and mind exercises, while playing the game, self-regulation skills are expected to improve. The game includes a Teachable agent (TA), based on a learning-by-teaching methodology [53, 54], encouraging the child to teach the TA early math. The game design and narrative provide rich multimodal feedback motivating the child for embodied interaction with the digital tablet involving affect, cognition and action [55].
3. Teachers scaffold the children’s participation throughout the sessions, supporting self-regulation in terms of focused attention, metacognition and emotional regulation, as well as providing support to solve the mathematical tasks and handle the digital device.

Preschool staff was trained before the start of the intervention for four 2-h evening sessions. The training included theoretical and practical elements, such as the rationale for learning early math before school start, the function of self-regulation and the role of the teacher in terms of scaffolding the child’s learning. Detailed descriptions of the exercises were reviewed and practiced, and the teachers spent time learning to use Magical Garden. Time was also spent planning the implementation of the intervention as part of the daily preschool schedule.

DIL was implemented for 1 h 5 times per week for 6-weeks intervention. Each session began with a group activity where the preschool staff taught the children about “the learning body” in terms of how the brain works, focused attention, breathing and meta-cognitive strategies for enhancing self-regulation. Different aspects of the learning body were trained through specific body exercises such as breathing, balancing a beanbag and focusing attention while being distracted. During 15–30 min per session the children individually played Magical Garden, in total adding up to a minimum of 20 sessions for each child, including at least 15 min effective interaction with the Magical Garden each time. In order to avoid distractions and enhance the individual nature of the activity the children wore headphones.

#### Control

The children at the control preschool units had business as usual during the 6 intervention weeks. However, in order to motivate preschool staff at the control units, this group was given BRUK, a self-evaluation tool developed by the Swedish National Agency for Education [2]. BRUK is adapted for different levels of the school system and the version used applies to the preschool setting. The tool is divided into four areas of investigation: 1) Each preschool’s development; 2) Norms, values, and influence; 3) Knowledge, development, and learning; and, 4) Transit, collaboration, and the surrounding world. Each area includes a number of indicators, and each indicator contains criteria that the preschool staff has to evaluate in relation to the work at the preschool. Examples of criteria are: criteria for goal fulfilment; criteria for implementation; criteria for operational conditions.

The work with BRUK was administered by the preschools themselves. A head of school introduced the tool to all staff at the control preschool units. Two areas were chosen to focus on in particular: “the learning environment”, and “routines at the preschool”. The areas and indicators are listed in an on-line formulary, with rating scales indicating Agree completely – Agree mostly – Agree to some extent – Do not agree. The pedagogical staff filled in a form of their own, before having discussions at group level. As areas of improvement were identified the group continued with elaborating methods to improve the quality with the aid of an experienced external preschool teacher. Six months later, a follow-up BRUK was conducted where the improvements were evaluated.

### Adherence

For the SEMLA intervention, implementation fidelity was monitored continuously. After each session the teachers documented which children had participated, whether they had worked mostly together with another child/children or alone, which activities had been undertaken, and whether anything out of the ordinary had occurred. At each preschool unit, one researcher supported and supervised the implementation during regular visits once per week. At these occasions, the sessions were video-recorded, providing rich data capturing the quality of the intervention implementation. Children were encouraged to participate, but were always allowed to opt out or discontinue participation at any time.

For the DIL intervention, objective adherence was registered by the software in terms of the amount of time a particular child had spent playing the game. Once a week, sessions were also recorded by a researcher, providing additional information about the participation of the individual children. During the entire intervention, program support for Magical Garden was provided by the researchers in the Education Technology Group in Lund in order to fix bugs and other potential problems related to the software. The software was upgraded continuously as problems were identified. Children were encouraged to participate, but were always allowed to opt out or discontinue participation at any time.

For the control group, adherence to the BRUK self-evaluation was not monitored other than by the preschools themselves.

### Preschool quality

Preschool quality was estimated on the basis of the ECERS-3 [56]. The rating scale measures process quality in the interactions between staff and children. This is assessed primarily through observation by trained and accredited observers/raters, who in this case were hired from the University of Gothenburg. The Early Childhood Environment Rating Scale is an internationally established tool for measuring preschool quality developed on comprehensive and global definitions of quality and have been found to be more predictive of children’s learning than structural factors such as group size, staff to child ratio, and costs [57]. A quality program must provide a satisfying degree of protection of health and safety, building positive relations, opportunities for stimulation and learning from experience http://ers.fpg.unc.edu/. The third edition consists of 35 items organized into 6 subscales: Space and furnishing, personal care routines, language and literacy, learning activities, and interaction. These are rated from 1 to 7. Minor adaptions of the ECERS-3 scale have been made to the Swedish context, which do not affect international comparison on the whole.

### Data collection

Pretest measurements were collected during a two-week period prior to the intervention period, and posttest measurements during a two-week period directly following the intervention period. Data collection consisted in behavioural testing, on the one hand, and participation in the ERP-experiment, on the other. Only a sub-sample of the children conducted the ERP-experiment.

### Behavioural testing

The behavioural testing was conducted by trained research assistants employed in the project.

Each child was tested twice during the pretest period and twice during the posttest period. Each session lasted between 20 to 40 min. The testing sessions were video-recorded in order to i) allow for validation of testing procedure, and ii) give interactional data on verbal and nonverbal behaviour as the child interacted with the test leader. The tests performed during the first session with each child were, in the order of testing: Dimensional Change Card Sorting (DCCS); Test of Emotional Comprehension (TEC); Bus Story (pretest)/Frog Story (posttest); Maths test; Head, shoulder, knees, toes (HSKT). At the second session, usually taking place the following day, the tests performed were: The Flanker Fish Task; What’s Wrong Cards; Peabody Picture Vocabulary Test (PPVT); Digit Span. The order of the tests was chosen to keep the two sessions equal in length, test different abilities and end sessions with more enjoyable tests (based on opinions of children in a pilot study), potentially leaving a memory of a fun experience for the children. The identical set-up for tests and order of tests were applied at the posttesting period except for two of the language tests: the narrative retell-test exchanged Bus Story to Frog Story and the three What’s Wrong Cards used at pretesting were exchanged to different ones for the posttesting. In both cases, the reason for change was to avoid the child remembering verbatim parts of the story/picture already heard and told.

### The ERP experiment

To measure auditory selective attention, we used a dichotic listening paradigm adapted to children [58] in which auditory test probes are embedded in two different stories, one attended and one unattended. We created a Swedish version of this paradigm called Swedish AUDAT. Test probes were linguistic, the syllable “Ba”, or non-linguistic, a ‘Bz’-like noise created by scrambling short segments of the linguistic probe. Approximately 500 probes were presented while the children attended stories. The ERP responses to probes were later compared between those embedded in attended and unattended stories (see outcome measures). The experiment was conducted by two researchers from the project and was carried out on site in a quiet room at the preschools using a mobile lab setup. EEG was recorded with an Active-two amplifier (BioSemi, Amsterdam, Netherlands) using 16 head channels in a cap, and 6 external channels (mastoid reference electrodes, and electrodes monitoring blinks and eye movement). Participating children had been oriented about the experiment and equipment previously. They were greeted and seated on a small chair where the cap and external electrodes were applied. They were instructed to pay attention to one of two simultaneous played stories presented via speakers. Pictures from the attended story were displayed on a laptop. Each recording session consisted of two story pairs. After each story, the children were asked questions about the attended story to ensure that they did attend. Each session lasted approximately 30–40 min.

### Outcome measures

In the study, we used multiple measures of the children’s attention/executive functioning skills, on the one hand, and their language and communication skills, on the other. These individual measures form the basis for composite measures of executive functioning, and language and communication, respectively. Early maths skills and socioemotional skills, on the other hand, were measured with two individual measures. In the following section, we describe the individual outcome measures and then the composite measures. We then present the background variables of the study.

#### Selective attention

Selective attention was measured using the Swedish AUDAT paradigm, as differences between ERP-responses to attended and unattended probe sounds. In the paradigm the following variables were manipulated (in order of importance): 1. Attending story to the left or right according to instructions and as a function of this also attending probes to the left or right. 2. Linguistic content in probes (“Ba” versus “Bz”). 3. Pause length between probes (200 ms, 550 ms, 1000 ms). 4. Characteristics of attended and unattended story (story content, story voice). To enhance contrast, story pairs played simultaneously always consisted of one female voice and one male voice. The order of probes and pauses was randomized. The order of attention direction, and selection of voices and stories was randomized and balanced over participants.

#### Measures of executive functioning

The components of executive functions can hardly be assessed in isolation, since the targeted cognitive process must be embedded in a certain task context that is likely to trigger other executive functions [16]. Children’s executive functions were therefore assessed using the following battery of tests: 1) The Dimensional Change Card Sort (DCCS), which mainly assesses the child’s cognitive flexibility [59, 60], 2) The Flanker Fish Task [40, 61, 62], which mainly assesses the child’s ability to suppress responses that are inappropriate in a particular context, 3) The Head-Shoulders-Knees-and-Toes (HSKT) test [63], which reflects the child’s ability to inhibit dominant responses of imitating the examiner and also puts high demands on working memory and focused attention, and 4) Forward and Backward Digit Span [64] which assesses short-term memory and working memory. The DCCS and the Flanker task were delivered via a tablet, but verbal instructions were given by the examiner, since there is no available Swedish-speaking tablet version. Results from a recent meta-analysis of DCCS indicate that the test format should not matter [59].

#### Measures of language

In order to assess the children’s receptive vocabulary skills, we used The Peabody Picture Vocabulary Test (PPVT) [65]. The PPVT has not been standardized for Swedish, but is widely used in Sweden, both clinically and in research. The lack of standardization entails that the psychometric qualities of the test are unclear. Hence, only raw scores were used for analysis. In addition to the PPVT test, the following measures related to language were also derived from children’s narratives: 1) lexical diversity (type:token ratio), 2) information score, i.e. how many events the child included in the narratives, 3) syntactic complexity, defined as number of subordinate clauses, 4) morphological complexity, defined as amount of well-formed utterances, and 5) text length, defined by total number of clauses [66].

#### Measures of communication

Communication was defined as the ability to interact in an age-adequate manner. The following behaviours were rated as either 0 (not adequate behaviour for the child’s age) or 1 (adequate behaviour for the child’s age): gaze (meeting eyes while speaking, following gaze and pointing instructions from the tester), gestures (use of complementary and/or supplementary gestures to convey or clarify verbal utterances), body posture (an at-ease appearance rather than fidgeting on the chair etc.), fluency/prosody (speaking up rather than whispering or holding objects/hands to the mouth), following instructions (cooperative versus uncooperative behaviour), turn-taking (adequate turn-taking behaviour rather than interruptions, silence, etc.), and initiative/curiosity (whether the child takes his or her own initiative in the interaction or not). The ratings were done by a rater watching 6 min of interaction from the posttest sessions, 2 min of introduction to DCCS, 2 min of story retell, and 2 min of HSKT. The rater was blind to the intervention of a specific child. The scores for the 7 behaviours were combined and divided by the maximum score of 21 (Tonér S & Gerholm T, Language and executive functions in Swedish preschoolers: a pilot study, submitted).

#### Measures of early maths skills

Children’s early maths skills were measured with an adapted version of the Number Sense Screener [67]. This instrument assesses aspects of early mathematic ability, namely one-to-one correspondence, number sense cardinality, ordinality and subitizing, which are some of the important mathematical concepts that develop during preschool years [68, 69].

#### Measure of emotional comprehension

The Test of Emotion Comprehension (TEC) was used to quantify the children’s emotional comprehension skills. The test assesses nine domains of emotional understanding: the recognition of emotions based on facial expressions, the comprehension of external emotional causes, the impact of desire on emotions, emotions based on beliefs, memory influence on emotions, the possibility of emotional regulation, the possibility of hiding an emotional state, having mixed emotions, and the contribution of morality to emotional experiences [70]. The TEC has been validated on Italian, Norwegian, Brazilian, Peruvian and Portuguese children [70, 71].

### Composite measures

Three composite measures were used to assess general EF, language ability, and communicative ability. These composite measures were calculated by summing the standardized individual component scores, and then standardizing the resulting sum scores. The EF composite measure consists of the individual DCCS, Flanker, HSKT, and FDS components, the language composite measure consists of the individual PPVT, Predicates, Subordinates and Events components, and the communication composite score consists of the individual TEC, and the scores from a blind rater on communicative skills measured from the video-recordings of the test situation.

### Background measures/covariates

#### Socioeconomic status (SES)

A 10-grade scale of socioeconomic status was estimated on the basis of both of the two caretakers’ annual income and their education level. The annual income of each caretaker was classified on the three-level income scale 1: 0–200,000 SEK, 2: 200000–500,000 SEK, and 3: > 500,000 SEK. Each caretaker’s education level was classified on a four-level scale 1: elementary school only, 2: upper secondary school, 3: vocational education, and 4: college/university education. A composite score on a scale from 0 to 10, consisting of even numbers only, was calculated for each parent p on the basis of their annual income score *Ip* and their education level score *Ep* in the following way:

$$ SESp=\left({\left( Ip+ Lp\right)}^{\ast }\ 2\right)-4 $$

For children living with both caretakers, the mean of both caretakers’ composite scores was used (thereby making it possible for the scale to include odd numbers). For children living with only one of the two caretakers, the score of that caretaker was used.

#### SCDI

The Swedish Communicative Development Inventories (SCDI) was used to assess aspects of the language abilities of the children [72, 73]. SCDI is the Swedish version of the MacArthur Communicative Development Inventories (CDI). The SCDI instrument assesses communicative and language abilities in children aged 8–48 months by means of parental reports. For this study, a preliminary version of SCDI – SCDI III [see [74, 75], for a validation of SCDI III] developed for children aged 30 to 48 months was used. This questionnaire includes questions pertaining to the children’s general language ability, vocabulary, grammatical ability, pronunciation, and meta-linguistic ability. In the assessment of the children’s vocabulary, parents are probed on their children’s word knowledge within the four semantic domains of food, body parts, thought, and emotion. The assessment of the children’s grammatical ability includes questions regarding their use of the past tense, the passive voice, conjunctions, and the use of comparative inflection (e.g., little, more, most). The total number of words that the child knows, as reported by their caregivers, serves as an estimate of the children’s’ vocabulary size (SCDI words). The ability of the children to form the past tense, to use the passive, and to use comparative inflection, as reported by their caregivers, was used as a score of the children’s morphological ability (SCDI morphology).

#### Age

As age can be expected to be highly predictive of all of the outcome measures, the age of the children in months was used as a control predictor.

#### Sex

A number of studies indicate a difference between boys and girls in the 4- to 5-year age span with respect to attention span [76], cooperation and social interaction skills [35, 36, 77], as well as command of language [78]. Sex has also been reported as a variable to consider in behavioural ratings, including hyperactivity, which is more frequently found or diagnosed in boys than in girls [79–81], and emotional difficulties such as phobia and eating disorders, which are more frequent in girls [82, 83]. These differences have not yet been documented in the Swedish preschool population, but sex was included as a control predictor on the basis of the studies mentioned above.

#### Preschool start

Preschool start might have an influence on some or many of the outcome measures. In a review of key studies from Europe, North America and Asia, Burger [83] concludes that an early start in preschool may have positive effects on child development, however, the findings are not conclusive. Loeb et al. [84] showed that academic gains from attending preschool in the context of the Head Start program in the United States are greater for those children who start preschool at the age of 2–3 than at a younger or older age. An early preschool start might either have a positive or negative effect on the children’s development. The age in months at which the children started in preschool was therefore included as a control predictor.

#### Preschool time

It might also be the case that the amount of time the children spend in the preschool has either a negative or a positive influence on their development. Burger [83] concludes that there is not enough evidence to draw conclusions regarding the ideal intensity of preschool participation. The above-mentioned study undertaken by Loeb et al. [84] showed that more hours in preschool led to higher academic gains but had some negative effects on behaviour. In order to investigate the contribution of preschool time in the present study the average number of preschool hours per week was included as a control predictor.

#### Second language

There are studies indicating that speaking a second or more language/s might have a positive effect on EF and inhibitory control [85–87]. Research indicates that bi- or multilingual children have both/all languages activated and potentially competing for selection [88, 89]. The control mechanisms required to inhibit the not targeted languages has been argued to influence and enhance the child’s inhibitory cognitive systems in a broader sense, aiding the individual in cognitive inhibitory functions more generally compared to monolingual children and adults [90]. Children learning more than one language, may, on the other hand, be somewhat delayed in their first language during the first few years of life [91]. The time when a second or third language is introduced affects the child’s results on executive functions tests with an advantage for younger bilinguals over children acquiring their second language at a later age [92]. Whether or not the child at hand speaks a second or third/fourth language (in addition to Swedish as their first language) was therefore included as a control predictor.

#### Swedish-as-L2

A number of children had a foreign language as their first language (L1), making Swedish their second (or perhaps even their third) language. As these children can be expected to be less proficient in Swedish, having Swedish as a second language was included as a control predictor.

#### Known language and/or other developmental disorders

Since all children who could participate in the behavioural testing were seen as eligible for participation in the study, any known/documented language disorder and/or other developmental disorder was included as a control predictor. The presence of developmental difficulties has been shown to significantly predict language development (e.g. [93]).

#### Family history of language disorders

This control predictor indicates whether there is a family history of language disorders, such as dyslexia. Language and literacy disorders are highly heritable and can also influence the child’s home language environment [93, 94].

#### SDQ

The Strengths and Difficulties Questionnaire (SDQ) is a parental report inventory behavioural screening for children aged 2 to 17 years old [95–97] that has been translated into a vast number of languages. The Swedish version targets the ages 3 through 16 and was translated by Smedje et al. [98]. The questionnaire contains 25 questions addressing behaviour in the Difficulties domain such as hyperactivity and attentional issues, behavioural deviations, difficulties with social relations and self-regulation skills concerning emotions. In the Strength domain, the questionnaire measures prosocial behaviour such as generosity, considerateness and the ability to wait for one’s turn. The SDQ has been validated for Swedish children from 6 to 10 years of age [99], which is outside the age span for the sample investigated here (4- to 6-year olds). The test is sensitive enough to distinguish between control and clinical samples, but is better adjusted for boys than for girls in that the majority of behavioural difficulties captured are within the hyperactivity scale, and hyperactivity is more frequently found in boys. The test misses some of the behavioural difficulties more frequently found in girls (e.g. emotional disorders like phobia and eating and anxiety disorders).

Both parental and preschool teacher SDQ assessments were obtained. On the basis of the scoring principles at the youth-in-mind SDQ website http://www.sdqinfo.com/, SDQ scores of prosocial behaviors as well as of total difficulties were calculated both for the parental and preschool teachers. Finally, composite scores consisting of the mean of the parental and the preschool teaching assessments were calculated for use in the statistical analyses.

#### Fidelity

As described in more detail above, fidelity measures in the SEMLA intervention were estimated on the basis of the documentation provided by the preschool teachers. After each session, teachers documented which children had participated actively in the SEMLA activity. For SEMLA, the fidelity score is the standardized number of sessions each child participated in.

In the DIL intervention, fidelity measures were provided both through teacher documentation, and through registration by the Magical Garden software of the number of sessions and amount of time spent playing the game. Teachers documented the number of times each child participated in “the learning body” sessions, and the Magical Garden software recorded the number of times each child played the game and the duration of each session. The fidelity score for the DIL intervention was calculated as the standardized sum of the number of “the learning body” sessions and the number of Magical Garden sessions, weighted with respect to the mean play time of the child.

For participants in the control group, a fidelity score of zero was used. This resulted in a standardized fidelity score with a mean of zero and a standard deviation of 1, zero being treated as a baseline value (i.e., as in the case for the participants in the control group, who did not participate in any intervention activities).

#### ECERS-3

As discussed above, the ECERS-3 consist of several measures of preschool quality relating to the quality of the preschool facilities, the daily routines, the use of language at the preschool, the learning environment, and the way that interactions take place [56]. In the statistical analyses, the standardized means of these individual scores were included as a control predictor at the preschool unit level.

### Statistical analysis

Statistical tests will be conducted in order to test the hypotheses outlined in Table 1. Different tests will be employed to test the hypotheses of each corresponding research question (RQ1 to RQ7). In the following section, the analyses for each corresponding research question are described.

### RQ1 analyses: Intervention effects

For RQ1 hypotheses, mixed effects modelling will be used to test the null hypotheses that there are no intervention effects on each of the respective outcome variables. Mixed effects modelling allows for the inclusion of multiple hierarchically nested random effects, such as children nested within preschool units and preschool units nested within preschools (e.g., [100]). The mixed effects model can thereby account for systematic influences of preschool units or preschools on the outcome variables. The model formula for the RQ1 hypothesis analyses is:

$$ {\mathrm{POSTSCORE}}_{\mathrm{ijk}}=\kern0.5em {\upalpha}_{0\mathrm{jk}}+{\upalpha}_{00\mathrm{k}}+{\upbeta}_1{\mathrm{INTERVENTION}}_{0\mathrm{jk}}+{\upbeta}_2{\mathrm{Y}}_{\mathrm{ijk}}+{\upbeta}_3{\mathrm{PRESCORE}}_{\mathrm{ijk}}+{\upbeta}_4{\mathrm{FIDELITY}}_{\mathrm{ijk}}+{\upbeta}_5{\mathrm{ECERS}}_{00\mathrm{k}}+{\upvarepsilon}_{\mathrm{ijk},}\ {\upvarepsilon}_{\mathrm{ijk}}\sim \mathrm{N}\left(0,{\upsigma^2}_{\upvarepsilon \mathrm{ijk}}\right),{\upalpha}_{\mathrm{j}}\sim \mathrm{N}\left(0,{\upsigma^2}_{\upalpha \mathrm{j}}\right),{\upalpha}_{\mathrm{k}}\sim \mathrm{N}\left(0,{\upsigma^2}_{\upalpha \mathrm{k}}\right) $$

In the models, the *i*th child is nested within the *j*th preschool unit, which in turn is nested within the *k*th preschool.

These models predict the postscore of the given outcome measure for child *i* as a function of the intervention administered at preschool unit *j*, using the control intervention as the baseline category. They also control for the set of background variables Y of child *i*, as well as the prescore measure of child *i*, centered around zero, as well as the fidelity score for child *i*, as explained in more detail above. Further, they control for the total ECERS rating of preschool *k*, thereby taking into account any influence of preschool quality on the outcome variables. Finally, they include individual intercepts α for each preschool *k*, as well as individual intercepts α for each preschool unit *j*, thereby controlling for systematic differences between preschool units and preschools with respect to the outcome variables.

Separate models will be conducted on selective attention, early maths skills and emotional comprehension, as well as on the composite measures of EF, language and communication.

### RQ2 analyses: Intervention differences

Hypotheses regarding intervention differences are tested using planned comparisons, comparing the postscore outcome measures in the SEMLA and the DIL interventions to each other.

### RQ3 analyses: Mediating effects

Hypotheses regarding mediating effects are tested on the basis of the method proposed by Preacher & Hayes [101]. Preacher & Hayes [101] differentiate between the *total effect* of X on Y, which is the effect when not taking into account the mediator M, the *direct effect* of X on Y, the effect when the mediator is taken into account, and the *indirect effect* of X on Y, the effect of X on Y as mediated by M. In order to estimate the indirect effect, Preacher & Hayes [101] propose a method of fitting the following three regression models:

Y_i_ = α + cX_i_ + ε_i_ where *c* estimates the total effect

M_i_ = α + aX_i_ + ε_i_ where *a* estimates the effect of X on the mediator M

Y_i_ = α + c’X_i_ + bX_i_ + ε_i_ where *c’* estimates the direct effect of X on Y and *b* the effect of M on Y when controlling for X

A prerequisite for a mediation effect is that c is significant (that is, that there is an effect of X on Y at all, that a is significant (that is, that there is an effect of X on M), and that b is significant (that is, there is an effect of M on Y over and above that of X).

The indirect effect, that is, the mediation effect, is equal to the product of a and b, *ab*, which in most cases is equivalent to the difference between c and c’. Several ways of calculating the standard error of ab have been proposed (e.g., [102, 103]; and see [104], for a review). However, following Preacher & Hayes [101], the standard error and significance of ab is calculated on the basis of bootstrapping. Here, ab is calculated on the basis of 10,000 bootstrap samples, yielding a distribution of 10,000 ab estimates. The point estimate of ab is simply the mean of that bootstrapped distribution, the standard error is the standard deviation of the distribution, and the confidence interval is the 5 and 95% percentile of the distribution. The bootstrapped *p* value of ab is the proportion of ab estimates that is equal to or lower than zero and the total number of ab estimates.

In order for a null hypothesis of no mediation effect to be rejected, the effects of a, b, c and ab, as estimated on the basis of bootstrapping, need to be significant.

### RQ4 analyses: Moderating effects

RQ4 hypotheses concern moderating effects, that is, hypotheses regarding whether the strength of an observed intervention effect on an outcome variable depends on another variable. In other words, RQ4 hypotheses involve hypotheses regarding interaction effects between interventions and other variables. These hypotheses are also tested with mixed effects modelling.

Firstly, we hypothesize that both the SEMLA and DIL intervention effects on selective attention, EF score, Language score and TEC will be negatively moderated by SES. That is, the intervention effects should be stronger on low-SES children than they are on high-SES children. These negative SES moderation hypotheses are tested with the model

$$ {\mathrm{POSTSCORE}}_{\mathrm{ijk}}=\kern0.5em {\upalpha}_{0\mathrm{jk}}+{\upalpha}_{00\mathrm{k}}+{\upbeta}_1{{\mathrm{INTERVENTION}}_{0\mathrm{jk}}}^{\ast }\ {\upbeta}_2{\mathrm{SES}}_{\mathrm{ijk}}+{\upbeta}_3{\mathrm{PRESCORE}}_{\mathrm{ijk}}+{\upvarepsilon}_{\mathrm{ijk},}\ {\upvarepsilon}_{\mathrm{ijk}}\sim \mathrm{N}\left(0,{\upsigma^2}_{\upvarepsilon \mathrm{ijk}}\right),{\upalpha}_{\mathrm{j}}\sim \mathrm{N}\left(0,{\upsigma^2}_{\upalpha \mathrm{j}}\right),{\upalpha}_{\mathrm{k}}\sim \mathrm{N}\left(0,{\upsigma^2}_{\upalpha \mathrm{k}}\right) $$

These models predict the postscore for child *i* as a function of the interaction between the intervention at preschool unit *j* and the SES score for child *i* while controlling for the prescore measure of child *i*, centered around zero. They also include individual intercepts α for each preschool *k* and for each preschool unit *j*, thereby controlling for differences between preschool units and preschools.

Secondly, both the SEMLA and DIL intervention effects on EF are hypothesized to be negatively moderated by the EF score at pretesting. In other words, the intervention effects on EF should be stronger on low-EF children than on high-EF children. This negative EF moderation hypothesis is tested with the model

$$ \mathrm{EF}\_{\mathrm{POSTSCORE}}_{\mathrm{ijk}}=\kern0.5em {\upalpha}_{0\mathrm{jk}}+{\upalpha}_{00\mathrm{k}}+{\upbeta}_1{{\mathrm{INTERVENTION}}_{0\mathrm{jk}}}^{\ast }\ {\upbeta}_2\mathrm{EF}\_{\mathrm{PRESCORE}}_{\mathrm{ijk}}+{\upvarepsilon}_{\mathrm{ijk},}\ {\upvarepsilon}_{\mathrm{ijk}}\sim \mathrm{N}\left(0,{\upsigma^2}_{\upvarepsilon \mathrm{ijk}}\right),{\upalpha}_{\mathrm{j}}\sim \mathrm{N}\left(0,{\upsigma^2}_{\upalpha \mathrm{j}}\right),{\upalpha}_{\mathrm{k}}\sim \mathrm{N}\left(0,{\upsigma^2}_{\upalpha \mathrm{k}}\right) $$

in which the EF postscore for child *i* is predicted on the basis of the interaction between the intervention at preschool unit *j* and the EF prescore of child *i*. It also includes individual intercepts α for each preschool *k* and each preschool unit *j*.

Thirdly, the SEMLA intervention effects on selective attention, EF score, math, TEC, communication score and the language score are expected to be positively moderated by the total ECERS score. In other words, there should be a stronger effect of the SEMLA intervention in high-quality preschools as estimated by the total ECERS score. These positive ECERS moderation hypotheses are tested with the model.

$$ {\mathrm{POSTSCORE}}_{\mathrm{ijk}}=\kern0.5em {\upalpha}_{0\mathrm{jk}}+{\upalpha}_{00\mathrm{k}}+{\upbeta}_1{{\mathrm{INTERVENTION}}_{0\mathrm{jk}}}^{\ast }\ {\upbeta}_2{\mathrm{ECERS}}_{00\mathrm{k}}+{\upbeta}_3{\mathrm{PRESCORE}}_{\mathrm{ijk}}+{\upvarepsilon}_{\mathrm{ijk},}\ {\upvarepsilon}_{\mathrm{ijk}}\sim \mathrm{N}\left(0,{\upsigma^2}_{\upvarepsilon \mathrm{ijk}}\right),{\upalpha}_{\mathrm{j}}\sim \mathrm{N}\left(0,{\upsigma^2}_{\upalpha \mathrm{j}}\right),{\upalpha}_{\mathrm{k}}\sim \mathrm{N}\left(0,{\upsigma^2}_{\upalpha \mathrm{k}}\right), $$

which predicts the postscore of the *i*th child from the interaction between the intervention at preschool unit *j* and the total ECERS score of preschool *k*, while controlling for the prescore of child i. Again, these models include individual intercepts α for each preschool *k* and each preschool unit *j*.

### RQ5 analyses: Background - outcome relationships

RQ5 hypotheses concern relationships between outcome variables and background variables. These will be tested with mixed effects models with the structure

$$ {\mathrm{SCORE}}_{\mathrm{ijk}}=\kern0.5em {\upalpha}_{0\mathrm{jk}}+{\upalpha}_{00\mathrm{k}}+{\upbeta}_1{\mathrm{Y}}_{\mathrm{ijk}}+{\upbeta}_2{{\mathrm{INTERVENTION}}_{0\mathrm{jk}}}^{\ast }\ {\upbeta}_3{\mathrm{TIME}}_{\mathrm{ijk}}+{\upbeta}_4{\mathrm{FIDELITY}}_{\mathrm{ijk}}+{\upbeta}_5{\mathrm{ECERS}}_{00\mathrm{k}}+{\upvarepsilon}_{\mathrm{ijk},}\ {\upvarepsilon}_{\mathrm{ijk}}\sim \mathrm{N}\left(0,{\upsigma^2}_{\upvarepsilon \mathrm{ijk}}\right),{\upalpha}_{\mathrm{j}}\sim \mathrm{N}\left(0,{\upsigma^2}_{\upalpha \mathrm{j}}\right),{\upalpha}_{\mathrm{k}}\sim \mathrm{N}\left(0,{\upsigma^2}_{\upalpha \mathrm{k}}\right), $$

predicting the outcome scores of child *i* on the basis of the background variables Y of child *i*, the interaction between the intervention at preschool unit *j* and the time of testing (pre vs. post) of child *i*, the fidelity score of child *i*, and, finally, the ECERS-3 score of preschool *k*. These models will also contain individual intercepts α for each preschool *k* and each preschool unit *j*. As these models contain parameters for the Intervention × Time interactions, they too test whether there are any significant intervention effects on the outcome scores. A positive Intervention × Time intervention effect shows that there is a positive effect on the outcome score in the given intervention group over and above any increase in the outcome score from pretesting to posttesting. Crucially, they also show which of the background variables are positively or negatively correlated with the corresponding outcome variables, even when intervention effects and differences between pre- and posttesting are controlled for.

Separate models will once again be conducted on selective attention, early maths skills, emotional comprehension, as well as on the composite measures of EF, language and communication, respectively.

### RQ6 analyses: Background - background analyses

RQ6 hypotheses regarding relationships between background variables will be tested on the basis of the significance of Pearson or spearman correlation coefficients, depending on the types of variables at hand.

### RQ7 analyses: Intervention effect differences

The final set of hypotheses concern differences in any observed intervention effects. In particular, we hypothesize that the SEMLA and DIL intervention effects on a particular outcome measure will show greater variation for the SEMLA intervention than for the DIL intervention. These hypotheses will be tested by investigating whether the variance of the pre- and post-difference scores of the respective outcome measures significantly differ between the two intervention groups. This will be done using either Levene’s [105] or Brown and Forsythe’s [106] test of equality of variances.

### Adjusting *P*-values for multiple comparisons

When multiple hypotheses are tested with inferential statistics, the risk for a false positive - falsely rejecting a null hypothesis in favour of the alternative hypothesis - goes up with the number of tests that are conducted. In other words, the risk for at least one type I error increases above the chosen alpha level with the number of tests that are conducted. In order to control for this alpha inflation when conducting multiple testing, some kind of *p*-value correction method is often employed. For the present analyses, we will use p-value correction for the *p*-values of the tests testing RQ1 to RQ4 hypotheses.

P-value correction will be done on the basis of the method proposed by Benjamini and Hochberg [107]. Instead of controlling the so-called familywise error rate, which is the probability of *at least one* Type I error, this method adjusts p-values for the so-called false discovery rate. The false discovery rate is the *expected proportion* of Type I errors (i.e., “false discoveries”) in a set of tests. Adjusting p-values for the false discovery rate is therefore a less conservative method than adjusting them for the familywise error rate, but a sensible method in cases where many p-values from many different tests need to be controlled for, such as in the present case.

### Power analyses

Power analyses were conducted on the basis of simulated data that in turn was based upon bootstrapped data from the pilot study. In the pilot study, each of the two interventions was conducted in two individual preschool units, and a third unit was used as control. Pre- and posttesting was conducted in a similar manner as in the full study. In the following, we first describe the data simulation procedure and then move on to the actual power analyses.

### Data simulation

The data used in the power analyses is based upon semi-bootstrapping. The data is based upon bootstrapped data from the pilot data set. Additional random noise is added to data in order to create a greater deal of variability between each bootstrapped cluster. Crucially, the simulation procedure allows for the specification of intervention main effects on particular dependent variables, making it possible to calculate power of intervention effects under different effect sizes.

The original pilot data set suffers from a lot of missing data, especially in the pretest data set. In particular, there is no language data available in the pretest data. Missing data was therefore interpolated on the basis of regression modelling. This was done by regressing each dependent measure on the background variables and the other dependent measures available. Regression models for each dependent measure were then selected on the basis of backward elimination. These models were then used to predict missing data points. The three language measures (number of unified predicates, number of subordinates and number of events) in the pretest data set were completely interpolated from the posttest data set in this way.

The bootstrapping procedure uses with-replacement random sampling to sample 9 clusters from each intervention group in the pilot data (i.e., 27 clusters in total), each cluster consisting of 12 children. As a result, each bootstrap sample consisted of a total of 324 children. In the bootstrapping of ERP data, cluster sizes ranged from 3 to 7 children, resulting in an average ERP bootstrap sample size of 135.

In order to increase the variation between clusters and individuals, additional random noise within the range of ±0.4 standard deviations, sampled from a uniform distribution, was added to the continuous predictor variables. For ECERS-3 scores, random noise within the range of ±0.5 standard deviations was used. All adjusted variables were trimmed so that any values lying outside the possible range of the variable at hand (e.g., 1–10 for SES) were adjusted to either the minimum or maximum value, and integer variables, such as SES, were rounded to the nearest integer.

**Fig. 1:**
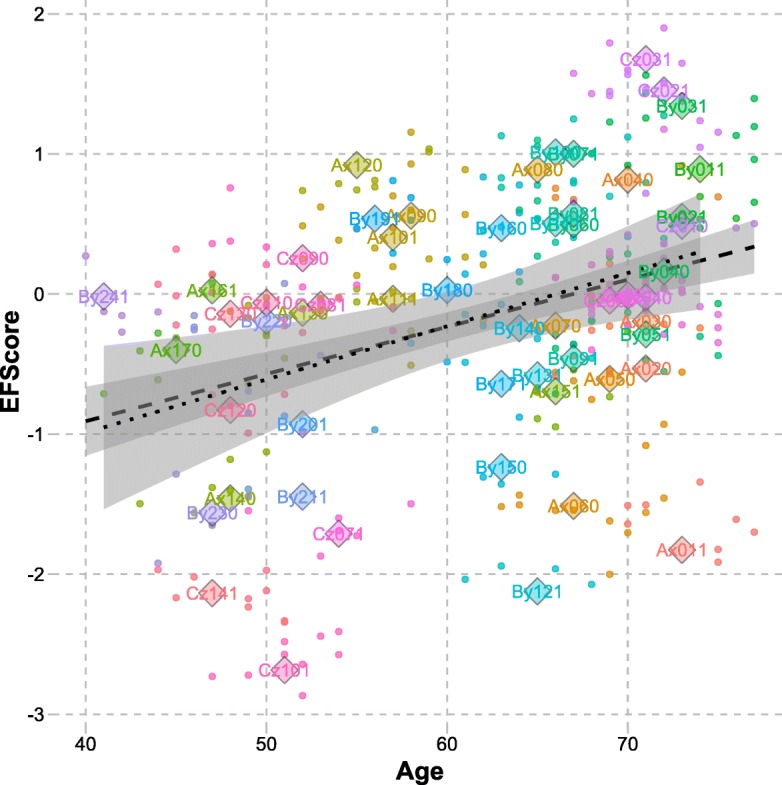
The relationship between EFScore and Age in the pilot data set and a bootstrapped data set with noise adjustment set to 0.5 standard deviations. Colours differentiate between children in the original pilot data. Diamonds illustrate data points in the pilot data set and dots show bootstrapped data points. The dashed regression line illustrates the linear fit in the bootstrapped data set, and the dotted line the pilot data set

Figure 1 illustrates the relationship between the pilot data and a bootstrapped data sample, in terms of plotting the relationship between EFScore and Age. The larger circles show the data points of the children in the pilot study (each child being colour coded), and the dots show the data points in the bootstrapped data. The dashed regression lines illustrate the linear fit between EF Score and Age in the bootstrapped data sets, and the dotted lines the linear fit between EF Score and Age in the pilot data set.

In addition to adding noise to the data, the dependent measures were adjusted for intervention main effects. This was done by adjusting the difference between the pre-, x_pre_, and post, x_post_ measurements of the aforementioned dependent measures so that they differed in strength in accordance with some adjustable effect size δ. More specifically, the mean difference between pre- and post-measurements was added to each post-measurement data point, i.e., x_post_ = x_post_ + Σx_pre_ / n_pre_ - Σx_post_ / n_post_, in order to ensure no initial difference between mean pre- and post-measurements. Then, the pooled standard deviation over interventions, σ, was calculated. Individual post-measurements were then increased in accordance with its effect size on the basis of the pooled standard deviation, i.e., x_post_ = x_post_ + δσ. Table 3 shows the effect size adjustments used for each intervention and each of the adjusted dependent measures. The table illustrates small, most likely insignificant, effects in the control intervention, strong EF effects but weak language and emotional comprehension effects in the DIL intervention, and, on the other hand, weak EF effects but strong emotional comprehension and language effects in the SEMLA intervention.

**Table 3:** Effect size adjustment δ for each intervention and dependent measure

Dependent Variable	Control	DIL	SEMLA
DCCSScore	0.1	0.5	0.3
FlankerScore	0.1	0.5	0.3
HeadShoulders	0.1	0.5	0.3
Math	0.1	0.5	0.3
FDS	0.1	0.5	0.3
TEC	0.1	0.2	0.6
PPVT	0.1	0.3	0.5
LangPredicates	0.1	0.3	0.5
LangSubordinates	0.1	0.3	0.5
LangEvents	0.1	0.3	0.5

### Power calculations

Power was calculated for each regression coefficient in all of the models described above, in the section about statistical analysis. This was done by refitting each model on 2000 bootstrap /simulation samples, generated as described above. Power statistics were then calculated on the basis of the resulting model coefficient distributions. We calculated mean beta values and also beta confidence intervals, consisting of the 5 and 95% quantiles of each beta coefficient distribution. A beta confidence interval that spans 0 indicates that the effect at hand is non-significant. We also calculated mean *p*-values for the respective coefficients over the 2000 model fits, p-value confidence intervals, consisting of the 5 and 95% quantiles of the distribution of these p-values, as well as bootstrapped p-values, calculated on the basis of the bootstrap distributions of model coefficients. More specifically, bootstrapped p-values are the proportions between the bootstrapped coefficients that span zero and the total number of coefficients (i.e., 2000). For a coefficient C with a mean beta value > 0, boot p-values are the proportion n(C = < 0) by N(C), and for a coefficient with a mean beta value < 0, boot *p*-values are the proportion n(C = > 0) by N(C). Further, the model coefficients that are critical for the hypothesis testing (e.g., the intervention effect coefficients) were adjusted for multiple comparisons, in terms of adjusting for the false discovery rate (see above).

Finally, power was calculated as the proportion of significant p-values (assuming alpha = 0.05) to the total number of 2000 p-values. In other words, power is the percentage of times that the bootstrapped models found the coefficient at hand to be significant.

In the following, we present power analysis statistics for all models testing hypotheses corresponding to research questions RQ1 to RQ5.

### RQ1 and RQ2 analyses: Intervention effects and intervention differences

Since RQ2 hypotheses regarding intervention differences will be tested on the basis of planned comparisons testing differences between the intervention effects in the RQ1 hypothesis models, RQ1 and RQ2 analyses will be tested together. As mentioned above, we will apply p-value correction to the p-values of coefficients that address the hypotheses.

Table 4 shows the statistics for the intervention model coefficients, including the planned comparisons of the intervention differences, for all RQ1 and RQ2 models. These models test intervention effects and intervention differences on EF, Language, Math and TEC. Apart for the SEMLA intervention effect on EF score and the DIL intervention effect on TEC, these analyses’ power show strong power for all intervention effects and intervention differences under the assumed effect sizes (see Table 3). Notably, the Beta coefficient for the SEMLA intervention on math is negative, indicating that SEMLA in fact might have a negative effect on early maths skills.

**Table 4:** Simulation statistics for the intervention model coefficients of all RQ1 and RQ2 models

Statistic	EFScore			Language Score			Math			TEC		
	DIL	SEMLA	DIL vs. SEMLA	DIL	SEMLA	DIL vs. SEMLA	DIL	SEMLA	DIL vs. SEMLA	DIL	SEMLA	DIL vs. SEMLA
Mean Beta	0.85	0.30	0.55	0.49	1.06	−0.57	0.10	−0.08	0.18	0.02	0.75	−0.73
Beta.lower	0.55	−0.03	0.22	0.21	0.78	−0.87	0.06	−0.14	0.11	−0.50	0.31	−1.18
Beta.upper	1.16	0.64	0.82	0.77	1.34	−0.28	0.13	−0.02	0.25	0.98	1.67	−0.31
boot.p	.000	.036	.000	.000	.000	.000	.000	.003	.000	.584	.002	.002
mean.p	.000	.096	.003	.008	.000	.003	.003	.029	.000	.424	.018	.022
p.lower	.000	.000	.000	.000	.000	.000	.000	.000	.000	.000	.000	.000
p.upper	.000	.774	.036	.080	.000	.033	.022	.326	.000	.971	.157	.195
Power	1.00	0.69	0.98	0.96	1.00	0.99	0.99	0.89	1.00	0.17	0.91	0.89
adj. p mean	.000	.118	.005	.010	.000	.001	.004	.038	.000	.472	.026	.031
adj. p.lower	.000	.000	.000	.000	.000	.000	.000	.000	.000	.000	.000	.000
adj. p.upper	.000	.816	.062	.130	.000	.050	.037	.408	.000	.975	.214	.252
adj. Power	1.00	0.61	0.97	0.94	1.00	0.97	0.98	0.86	1.00	0.16	0.87	0.85

### RQ3 analyses: Mediating effects

As described in more detail above, hypotheses regarding mediating effects are tested on the basis of the method proposed by Preacher & Hayes [101]. Below, we report power analysis statistics for the significance tests of the indirect effect ab from the mediation analyses. These statistics consist of bootstrapping statistics for the beta coefficients (means and confidence intervals), *p*-values (bootstrapped p-values, mean p-values and their confidence intervals), and power calculations. We also present adjusted p-value statistics as well as power calculations as based upon adjusted p-values. It should be stressed, that power calculations were done somewhat differentially than it was done for the coefficients in the mixed effects regression models. In these analyses, the indirect effect ab of a moderation analysis in a given bootstrap sample was deemed significant, firstly, if c, b and a were significant, and secondly, if ab - as estimated by the Sobel test - was significant. Power analyses were therefore conducted on the basis of the p-values of all of these parameters. Adjusted power is calculated on the basis of adjusted p-values of the Sobel test only, although it might be more accurate to also adjust the *p*-values of c, b and a in the power calculation.

Statistics of tests of the indirect effects are shown in Table 5. Apart for the EF mediation effects on the DIL and SEMLA interventions on TEC, these power analyses indicate strong power for all mediation effects under the assumed effect sizes shown Table 3. However, for the math mediation effect on the SEMLA intervention effect on EF (i.e., “EF-by-Math”), the Beta coefficient is negative. This is in line with the previous observation of negative Beta coefficient for the intervention effect of SEMLA on Math. This suggests that the SEMLA intervention effect has a negative impact on children’s early maths skills, which in turn leads to a negative math mediation effect of the SEMLA intervention on EF.

**Table 5:** Simulation statistics of the analyses of the indirect effects in the mediation tests, testing mediation effects of EF on the intervention effects on TEC (TEC-by-EF), Language (Language-by-EF), Math (Math-by-EF), the mediation effect of Language on the intervention effect on EF (EF-by-Language), and the mediation effect of Math on the intervention effect of EF (EF-by-Math)

Statistics	TEC-by-EF		Language-by-EF		Math-by-EF		EF-by-Language		EF-by-Math	
	DIL	SEMLA	DIL	SEMLA	DIL	SEMLA	DIL	SEMLA	DIL	SEMLA
Mean Beta	0.54	0.20	0.51	0.31	0.08	0.05	0.37	0.68	0.36	−0.59
Beta.lower	0.24	0.04	0.24	0.06	0.03	0.01	0.20	0.48	0.22	−0.90
Beta.upper	0.88	0.41	0.76	0.55	0.12	0.09	0.55	0.89	0.51	−0.31
boot.p	.000	.008	.000	.008	.000	.008	.000	.000	.000	.000
mean.p	.004	.064	.003	.052	.003	.052	.004	.000	.001	.001
p.upper	.000	.000	.000	.000	.000	.000	.000	.000	.000	.000
p.lower	.025	.533	.020	.525	.022	.525	.032	.000	.007	.001
Power	0.51	0.75	0.97	0.81	0.99	0.81	0.97	0.82	0.99	0.82
adjusted.p mean	.006	.082	.005	.067	.005	.067	.006	.000	.002	.001
adjusted.p.upper	.000	.000	.000	.000	.000	.000	.000	.000	.000	.000
adjusted.p.lower	.048	.620	.042	.620	.042	.620	.056	.000	.014	.003
adjusted.power	0.51	0.67	0.96	0.77	0.97	0.77	0.96	0.82	0.99	0.81

### RQ4 analyses: Moderating effects

As described above, RQ4 hypotheses concern moderating effects, that is, interaction effects between interventions and other variables. In Table 6, we report power analysis statistics for the significance tests of the critical interaction effects. These concern interaction effects between SES and the intervention effects on EF, Language, Math and TEC, as well as the interaction effect between EF score at pretesting and the EF posttest score. Again, we report bootstrapping statistics for the beta coefficients (means and confidence intervals), *p*-values (bootstrapped p-values, mean p-values and their confidence intervals), power calculations, as well as adjusted p-value statistics and power calculations based upon adjusted p-values. As can been seen in the table, moderating effects of SES on EF and Language have very high power under the assumed effect sizes. These moderating effects also show the expected direction in that all of the beta coefficients are negative. In other words, the intervention effects on EF and Language can be expected to be stronger for low-SES children than for high-SES children. The moderating effects of SES on TEC, on the other hand, show a different pattern. First, power for the SES moderation effect on the SEMLA intervention effect on TEC is low, indicating that no effect can be expected. Second, the beta coefficient of the SES moderation on the DIL intervention effect is positive, contrary to the hypothesis. In other words, this indicates that it is high-SES children, rather than low-SES children that will show a stronger intervention effect of SEMLA on TEC.

**Table 6:** Simulation statistics of analyses of moderating effects, in terms of interactions between SES and the intervention effects on EF, Language, and TEC, and the interaction between EF pretest score and the intervention effects on EF

Statistic	EFScore		LanguageScore		TEC		EFScore	
	DIL × SES	SEMLA × SES	DIL × SES	SEMLA × SES	DIL × SES	SEMLA × SES	DIL × EFpre	SEMLA × EFpre
Mean Beta	−0.54	−0.53	−0.79	−0.89	0.65	0.24	−0.23	0.30
Beta.lower	−0.74	−0.71	−0.96	−1.08	0.28	−0.09	−0.41	0.13
Beta.upper	−0.33	− 0.36	−0.61	− 0.70	1.05	0.59	−0.04	0.46
boot.p	.000	.000	.000	.000	.000	.083	.010	.000
mean.p	.000	.000	.000	.000	.022	.306	.114	.025
p.upper	.000	.000	.000	.000	.000	.002	.000	.000
p.lower	.003	.001	.000	.000	.185	.961	.677	.211
Power	1.00	1.00	1.00	1.00	0.89	0.23	0.54	0.87
adj mean.p	.001	.000	.000	.000	.032	.353	.144	.036
adj p.upper	.000	.000	.000	.000	.000	.004	.001	.000
adj p.lower	.006	.002	000	.000	.248	.969	.749	.275
adj power	1.00	1.00	1.00	1.00	0.84	0.18	0.47	0.82

### RQ5 analyses: Background-outcome relationships

Here, we present power analysis statistics for RQ5 analyses, concerning hypotheses regarding relationships between background variables and outcome variables. As described above, these will also be tested with mixed effects models, predicting each outcome variable as function of the background variables, the interventions and the intervention × test (pretest vs. posttest) interaction. As such, these models test the influence of each background variable on each outcome measure, as measured both at pre- and posttesting, while controlling for the influence of all other background variables as well as differences between intervention groups and intervention effects. The power analysis statistics for the background-outcome relationships are shown in Table 7. The table only shows statistics for tests of relationships that we have specific hypotheses for, including bootstrapping statistics for the beta coefficients (means and confidence intervals), *p*-values (bootstrapped p-values, mean p-values and their confidence intervals), power calculations. For tests of RQ5 hypotheses, we do not plan to conduct p-value correction; we therefore do not report p-value adjusted statistics.

**Table 7:** Simulation statistics of analyses of background-outcome relationships between EF, on the hand, and SES, Sex, and 2nd Language, on the other, between TEC, on the one hand, and Sex and 2nd Language, on the other, and between Math and 2nd Language, and finally between Language and SES

Statistic	EFScore			TEC		Maths	Language
	SES	Sex	2nd lang.	Sex	2nd lang.	2nd lang.	SES
Mean Beta	−0.07	−0.04	−0.04	−0.20	0.02	0.02	0.02
Beta.lower	−0.16	−0.23	−0.23	−0.50	−0.25	−0.02	−0.07
Beta.upper	0.02	0.13	0.13	0.12	0.31	0.06	0.11
boot.p	.058	.327	.327	.106	.436	.145	.329
mean.p	.213	.452	.452	.295	.510	.342	.440
p.upper	.000	.012	.012	.001	.024	.001	.008
p.lower	.911	.970	.970	.948	.974	.945	.978
Power	0.38	0.09	0.09	0.26	0.05	0.21	0.10

The table shows that the power is low, or virtually non-existent, for all of the included background-outcome relationships. The strongest relationship in terms of power is that between SES and EF. However, as shown by its Beta coefficient, here the power analysis predicts a negative relationship between SES and EF, low-SES children having a somewhat better EF ability than high-SES children, opposite to the hypothesis.

### Data management

Data is handled according to the regulations of Stockholm University. Video data is stored on three servers: one at the department of Child and Youth studies and two at the Linguistics department. All three are located in secure rooms. There are two Windows servers and one Linux server. Communication to these servers is secure, and they can only be accessed by members of the project. The servers are synchronized and backed up regularly. Raw data is kept encrypted and with password protection, and researchers in the team can upload files and videos but cannot change or download files. Processed files are kept separate from files being processed, and from unprocessed files. All involved researchers have access to files for processing and have signed a document stating their understanding of the ethical rules applying to sensitive data of the kind gathered for the project. Only the first author and the PI, Hillevi Lenz-Taguchi, have access to the code key which links codes to particular children and preschools.

### Dissemination

The main results of this study will be reported through one journal article (to be submitted to BMC Psychology or Journal of Trends in Neuroscience and Education). Additional journal articles will address the interventions in detail (to be submitted to Journal of Cognition and Development; Journal of Early Childhood Education Research) the EEG-paradigm used (to be submitted to Frontiers of Psychology), the collaboration set-up (to be submitted to Journal of Cognition and Development), the narrative development of preschool age children (to be submitted to Journal of Child Language) and the relation between EF and language in preschoolers (to be submitted to Early Childhood Research Quarterly).

## Discussion

This paper describes the design and implementation of an intervention RCT study, contrasting two pedagogical methodologies in terms of their effect on preschool children’s language, executive functions, attention, socioemotional skills and early maths skills. The main goal of the project was to investigate whether two pedagogical practices have different impacts on children’s learning, in contrast to a control condition (preschool business as usual). Further questions that were posed concerned in what manner, if at all, the different pedagogical practices had different impacts on children of different socioeconomical backgrounds and children of different ages or sexes.

The strength of the present project is in the collaboration between different research traditions and methodologies, which created an investigative net not often seen in educational science (or elsewhere). The participating researchers’ different academic backgrounds (psychology, linguistics, speech therapy, pedagogy, cognition), in theoretical as well as methodological practices, created a platform for discussion, investigation and research that enables gathering a broad spectrum of data and carrying out a wide array of analyses. Whereas most studies adopt either a qualitative or a quantitative approach, this project uses methodologies from both sides. Quantitative measures consist of results from standardized behavioural tests, the ERP-measure, and fidelity scores from tablets and adherence protocols. Qualitative measures are based on video-recorded interaction data, scales of narrative complexity stemming from transcriptions of child-tester interactions, and judgements of pedagogue-child interaction and preschool quality. The background data gathered in the present project – socioeconomic status, languages spoken, number of siblings, living conditions, health issues, etc. – enable analyses of correlations between variables not previously studied in the Swedish preschool context. The results from these data could play a significant role in how we understand children’s path through the educational system and what means we have to affect it in desirable ways. Through this rich research approach, the project carries the potential to contribute knowledge deeply needed in turning the preschool curriculum into one based on thorough scientific investigations.

The weaknesses connected to the project concern implementation of the study across preschools. Different engagement levels have been noted from different practitioners, and although the control preschools were enrolled in a self-evaluative assessment (BRUK) in the hopes of making them feel that they were actively involved in the study, there is a possibility that being assigned to the control condition in and of itself creates a sense of alienation from the project that could affect the outcome. The major obstacle observed throughout the project was, however, the different levels of participation found in different preschools as a result of particular individuals being more or less personally engaged in the tasks. Although this is a problem for a controlled study, it reflects the everyday situation of all educational practices. In the future, ways to measure and handle the “human aspect” of pedagogical reality as an influential factor in its own right need to be addressed. Thorough qualitative analysis of the interaction data from the present project could, in its continuation, contribute to a deepened discussion and knowledge of how we handle this influential but evasive reality.

The analysis methods chosen are ideal for analysing data sets such as the present one. In particular, by using mixed effects modelling it is possible to account for the hierarchical structure of the data, with children nested within preschool units, and preschool units nested within preschools. By including random slopes for each of these levels, the mixed effects models account for systematic variation that might exist between preschools or preschool units. The mixed effects model also allows for the inclusion of independent variables and covariates at different levels of nesting, such as the inclusion of interventions and the ECERS-3 scores on the preschool unit level, together with other covariates at the child level.

The results of the study are limited, containing outcomes from only 432 children of the approximately 500,000 attending Swedish preschools. However, the results carry the seed for a preschool setting based on evidence from a multifaceted investigation including considerations of children’s development through both measurable, quantitative test-scores on general abilities, and fine-grained qualitative analyses of skills not necessarily visible except through in-depth analyses of interactions between children and pedagogues.

## Additional file

Additional file 1: Letter to parents. (PDF 282 kb)

## Footnotes

1 The Magical Garden is developed in cooperation between Lund University and Stanford University, see http://portal.research.lu.se/portal/en/projects/the-magical-garden(47a4722c-0e85-4af2-bfdb-53059ad48184).html.

## References

[1] https://www.skolverket.se/statistik-och-utvardering/nyhetsarkiv/nyheter-2013/allt-fler-barn-i-forskolan-1.193605. Accessed 31 May 2018.

[2] Lpfö 89/10. https://www.skolverket.se/om-skolverket/andra-sprak/in-english. Accessed 7 Feb 2018.

[3] National Agency of Education, 362: 2011. https://www.skolverket.se/om-skolverket/andra-sprak/in-english/the-swedish-education-system/preschool-class/what-rules-govern-preschool-classes-1.72260. Accessed 31 May 2018.

[4] Kjällander S, Moinian F (2014). Digital tablets and applications in preschool–preschoolers’ creative transformation of didactic design. Designs for learning.

[5] Thorell LB, Lindqvist S, Bergman Nutley S, Bohlin G, Klingberg T (2009). Training and transfer effects of executive functions in preschool children. Dev Sci.

[6] Nemmi F, Nymberg C, Helander E, Klingberg T (2016). Grit is associated with structure of nucleus Accumbens and gains in cognitive training. J Cogn Neurosci.

[7] Black B, Logan A. Links between communication patterns in mother-child, father-child, and child-peer interactions and children's social status. Child Dev. 1995. 10.1111/j.1467-8624.1995.tb00869.x.

[8] Blair C, Raver CC (2015). School readiness and self-regulation: a developmental psychobiological approach. Annu Rev Psychol.

[9] Hoff-Ginsberg E (1991). Mother-child conversation in different social classes and communicative settings. Child Dev.

[10] Anders Y, Grosse C, Rossbach H-G, Ebert S, Weinert S (2013). Preschool and primary school influences on the development of Children's early numeracy skills between the ages of 3 and 7 years in Germany. Sch Eff Sch Improv.

[11] Ginsborg J, Clegg J, Ginsborg J (2006). The Effects of Socioeconomic Status on Children’s Language Acquisition and use. Language and Social Disadvantage: Theory into Practice.

[12] Sarsour K, Sheridan M, Jutte D, Nuru-Jeter A, Hinshaw S, Boyce WT (2011). Family socioeconomic status and child executive functions: the roles of language, home environment, and Single Parenthood. Journal of the International Neuropsychological Society.

[13] Hoff E (2003). The specificity of environmental influence: socioeconomic status affects early vocabulary development via maternal speech. Child Dev.

[14] Vallotton CD, Ayoub CA. Use your words: The role of language in the development of toddlers’ self-regulation. Early Childhood Research Quarter. 2010. 10.1016/j.ecresq.2010.09.002. NIHMS[235616]. 10.1016/j.ecresq.2010.09.002 PMC3184006 21969766

[15] Blain-Brière B, Bouchard C, Bigras N. The role of executive functions in the pragmatic skills of children age 4–5. Frontiers Pstchol. 2014. 10.3389/fpsyg.2014.00240. 10.3389/fpsyg.2014.00240 PMC3960491 24688480

[16] Miyake A, Friedman NP, Emerson MJ, Witzki AH, Howerter A, Wager TD. The unity and diversity of executive functions and their contributions to complex “frontal lobe” tasks: a latent variable analysis. Cogn Psychol. 2000; 10.1006/cogp.1999.0734 10.1006/cogp.1999.0734 10945922

[17] Miyake A, Friedman NP (2012). The nature and Organization of Individual Differences in executive functions: four general conclusions. Curr Dir Psychol Sci.

[18] Diamond A. Executive functions. Annu Rev Psychol. 2013; 10.1146/annurev-psych-113011-143750. 10.1146/annurev-psych-113011-143750 PMC4084861 23020641

[19] Lonigan CJ, Allan DM, Phillips BM. Examining the predictive relations between two aspects of self-regulation and growth in preschool children’s early literacy skills. Dev Psychol. 2017; 10.1037/dev0000247 10.1037/dev0000247 PMC5191909 27854463

[20] Posner M, Fan J, Pomerantz JR (2008). Attention as an organ system. Topics in integrative neuroscience: from cells to cognition.

[21] Posner MI. Attentional Networks and Consciousness. Front Psychol. 2012; 10.3389/fpsyg.2012.00064 10.3389/fpsyg.2012.00064 PMC3298960 22416239

[22] Clements DH, Sarama J, Germeroth C (2016). Learning executive function and early mathematics: directions of causal relations. Early Childhood Res Quarterly..

[23] Ridderinkhof KR, van der Stelt O. Attention and selection in the growing child: views derived from developmental psychophysiology. Biol Psychol. 2000; doi-org.ezp.sub.su.se/10.1016/S0301-0511(00)00053-3. 10.1016/s0301-0511(00)00053-3 11035220

[24] D’Angiulli A, Herdman A, Stepells D, Hertzman C. Children's event-related potentials of auditory selective attention vary with their socioeconomic status. Neuropsychology. 2008; 10.1037/0894-4105.22.3.293 10.1037/0894-4105.22.3.293 18444707

[25] Neville H, Stevens C, Pakulak E, Bell T, Fanning J, Klein S, Isbell E. Family-based training program improves brain-function, cognition, and behavior in lower socioeconomic status preschoolers. Proc Natl Acad Sci U S A. 2013; 10.1073/pnas.1304437110. 10.1073/pnas.1304437110 PMC3718115 23818591

[26] Cozolino L (2013). The social neuroscience of education.

[27] Siegel DJ (2012). The developing mind.

[28] Ochs E (1991). Socialization through language and interaction: a theoretical introduction. Issues in Applied Linguistics.

[29] Gerholm T. Socialization of verbal and nonverbal emotive expression in young children. Doctoral dissertation. Dept. of linguistics, Stockholm University; 2007.

[30] Gerholm T (2008). Att skapa ett språk i en kontext. Psyke Logos.

[31] McClelland MM, Acock AC, Piccinin A, Rhea SA, Stallings MC (2013). Relations between preschool attention span-persistence and age 25 educational outcomes. Early Child Res Q.

[32] Moffitt TE, Arseneault L, Belsky D, Dickson N, Hancox RJ, Harrington H, Houts R, Poulton R, Roberts BW, Ross S, Sears MR, Murray Thomson W, Caspi A. A gradient of childhoos self-control predicts health, wealth, and public safety. PNAS. 2011; 10.1073/pnas.1010076108. 10.1073/pnas.1010076108 PMC3041102 21262822

[33] Kroesbergen EH, Van Luit JEH, Van Lieshout ECDM, Van Loosbroek E, Van de Rijt BAM. Individual differences in early numeracy: the role of executive functions and subitizing. J Psychoeduc Assess. 2009; 10.1177/0734282908330586.

[34] Diamond A, Ling DS. Developmental Cognitive Neuroscience 2016; 10.1016/j.dcn.2015.11.005. 10.1016/j.dcn.2015.11.005 PMC5108631 26749076

[35] Durlak J, Weissberg RP, Dymnicki AB, Taylor RD, Schellinger KB (2011). The impact of enhancing Students' social and emotional learning: a meta-analysis of school-based universal interventions (PDF). Child Dev.

[36] Yoder N. Teaching the whole child instructional practices that support social-emotional learning in three teacher evaluation frameworks. Center on great teachers & leaders at American Institutes for Research. Revised edition. 2014. https://gtlcenter.org/sites/default/files/TeachingtheWholeChild.pdf. Accessed 6 Feb 2018.

[37] Morrison Gutman L, Schoon I. The impact of non-cognitive skills on outcomes for young people. Literature review 21. Institute of Education. University of London. 2013. https://v1.educationendowmentfoundation.org.uk/uploads/pdf/Non-cognitive_skills_literature_review_1.pdf. Accessed 23 Jan 2018.

[38] Payton JW, Wardlaw DM, Graczyk PA, Bloodworth MR, Tompsett CJ, Weissberg RP. Social and emotional learning: a framework for promoting mental health and reducing risk behavior in children and youth. J Sch Health. 2000; 10.1111/j.1746-1561.2000.tb06468.x. 10.1111/j.1746-1561.2000.tb06468.x 10900594

[39] Lenz Taguchi H, Palmer A. Dokumentation för lärande. Socioemotionellt och materiellt lärande i förskolan. In: Pramling N, Lindgren A-L, Säljö R. editors. Förskolan och barns utveckling. Malmö: Gleerups; 2017.

[40] Rueda MR, Checa P, LM C’m (2012). Enhanced efficiency of the executive attention network after training in preschool children: immediate changes and effects after two months. Dev Cogn Neurosci.

[41] Shin M-S, Jeon H, Kim M (2016). Effects of smart-tablet-based neurofeedback training on cognitive function in children with attention problems. J Child Neurol.

[42] Hillman CH, Pontifex MB, Castelli DM (2014). Effects of the FITKids randomized controlled trial on executive control and brain function. Pediatrics.

[43] Erickson KI, Hillman CH, Kramer AF (2015). Physical activity, brain, and cognition. Curr Opin Behav Sci.

[44] Wass S, Porayska-Pomsta K, Johnson MH (2011). Training attentional control in infancy. Curr Biol.

[45] Murray J, Theakston A, Wells A (2016). Can the attention training technique turn onemarshmallow into two? Improving children’s ability to delay gratification. Behav Res Ther.

[46] Haake M, Husain L, Anderberg E, Gulz A. No child behind nor singled out? – adaptive instruction combined with inclusive pedagogy in early math software. In: Conati C, Heffernan N, Mitrovic A, Verdejo M. editors. Artificial Intelligence in Education. AIED 2015. Lect Notes Comput Sci, vol 9112. Springer, Cham.

[47] Brain Development Lab at Oregon University, see: https://bdl.uoregon.edu/

[48] Chan AW, Tetzlaff JM, Altman DG, Laupacis A, Goetzche PC, et al. SPIRIT 2013 statement: defining standard protocol items for clinical trials. Annals of Internal Medicin. 2013; 10.7326/0003-4819-158-3-201302050-00583. 10.7326/0003-4819-158-3-201302050-00583 PMC5114123 23295957

[49] Schulz F, Altman G, Moher D. CONSORT 2010 statement: updated guidelines for reporting parallel group randomised trials. BMJ. 2010; 10.1136/bmj.c332 10.1136/bmj.c332 PMC2844940 20332509

[50] Campbell M, Piaggio G, Elbourne D, Altman G. Consort 2010 statement: extension to cluster randomised trials. BMJ. 2012; 10.1136/bmj.e5661. 10.1136/bmj.e5661 22951546

[51] Boutron I, Moher D, Altman D, Schultz K, Ravaud P (2008). Methods and processes of the CONSORT group: example of an extension for trials assessing nonpharmacologic treatments. Ann Intern Med.

[52] Hoffman TC, Glasziou PP, Boutron I, Milne R, et al. Better reporting of interventions: template for intervention description and replication (TIDieR) checklist and guide. BMJ. 2014; 10.1136/bmj.g1687. 10.1136/bmj.g1687 24609605

[53] Blair K, Schwartz D, Biswas G, Leelawong K. Pedagogical agents for learning by teaching: Teachable agents. Educ Technol*-*SADDLE BROOK THEN ENGLEWOOD CLIFFS NJ-, 2007;47;1;56.

[54] Husain L, Gulz A, Haake M (2015). Supporting early math: rationales and requirements for high quality software. J Comput Mathematics Sci Teach.

[55] Mascolo MF, Fischer KW. The dynamic development of thinking, feeling, and acting over the life span. The handbook of life-span development. I:6. 2010.

[56] Harms T, Clifford RM, Cryer D (2014). Early childhood environment rating scale, third edition (ECERS-3).

[57] Whitebook M, Howes C, Phillips D (1989). Who cares? Child care teachers and the quality of care in America: final report, National Child Care Staffing Study.

[58] Coch D, Sanders LD, Neville HJ (2005). An event-related potential study of selective auditory attention in children and adults. J Cogn Neurosci.

[59] Doebel S, Zelazo PD. A meta-analysis of the dimensional change card sort: implications for developmental theories and the measurement of executive function in children. Dev Rev. 2015. 10.1016/j.dr.2015.09.001. 10.1016/j.dr.2015.09.001 PMC4778090 26955206

[60] Zelazo PD, Anderson JE, Richler J, Wallner-Allen K, Beaumont JL, Weintraub S. Ii. Nih toolbox cognition battery (cb): measuring executive function and attention. Monogr Soc Res Child Dev. 2013. 10.1111/mono.12032. 10.1111/mono.12032 23952200

[61] Rueda MR, Posner MI, Rothbart MK. The development of executive attention: contributions to the emergence of self-regulation. Dev Neuropsychol. 2005. 10.1207/s15326942dn2802_2. 10.1207/s15326942dn2802_2 16144428

[62] Posner MI, Rothbart MK, Voelker P (2016). Developing brain networks of attention. Current Opinions Pediatrics.

[63] Cameron Ponitz CE, McClelland MM, Jewkes AM, Connor CM, Farris CL, Morrison FJ. Touch your toes! Developing a direct measure of behavioral regulation in early childhood. Early Childhood Res Quarterly. 2008. 10.1016/j.ecresq.2007.01.004.

[64] Gathercole SE, Baddeley A (1996). The Children’s Test of Non-Word Repetition.

[65] Dunn LM, Dunn LM (2007). Peabody Picture Vocabulary Test.

[66] Berman RA. On the ability to relate events in narrative. Discourse Processes. 1988. 10.1080/01638538809544714.

[67] Jordan NC, Glutting J, Dyson N, Hassinger-Das B, Irwin C. Building kindergartners' number sense: a randomized controlled study. J Educ Psychol. 2012. 10.1037/a0029018. 10.1037/a0029018 PMC4389641 25866417

[68] Charlesworth R, Leali S. Using problem solving to assess young Children's mathematics knowledge. Early Childhood Educ J. 2012. 10.1007/s10643-011-0480-y.

[69] Lundström M. Förskolebarns strävanden att kommunicera matematik. Doctoral Thesis, Faculty of Education. Göteborg: University of Gothenburg; 2015.

[70] Rocha A, Roazzi A, Lopes Da Silva A, Candeias A, Moita Minervino C, Roazzi M, Pons F. Test of Emotion Comprehension: Exploring the underlying structure through Confirmatory Factor Analysis and Similarity Structure Analysis. In: Roazzi A, Campello de Souza B. editors. Facet Theory: Searching for Structure in Complex Social, Cultural and Psychological Phenomena, Editora UFPE, Wolfgang Bilsky; 2015. p. 66–84. 10.13140/RG.2.1.2457.4483.

[71] Albanese O, Grazzani I, Molina P. Children's emotion understanding: Preliminary data from the Italian validation project of Test of Emotion Comprehension (TEC). In: Pons F, Daniel M-F, Lafortune L, Doudin PA, Albanese O. editors. Toward emotional competences. Aalborg University Press, Aalborg; 2006. p. 39–53.

[72] Berglund E, Eriksson M. Communicative development in Swedish children 16-28 months old: the Swedish early communicative development inventory—words and sentences. Scand J Psychol. 2000. 10.1111/1467-9450.00181. 10.1111/1467-9450.00181 10870432

[73] Eriksson M, Westerlund M, Berglund E. A Screening version of the Swedish communicative development inventories designed for use with 18-month-old children. J Speech Lang Hear Res. 2002. 10.1044/1092-4388(2002/077).. 10.1044/1092-4388(2002/077) 12381052

[74] Larsson A. Barns språkutveckling: Validering av SECDI-III mot CCC-2, Independent thesis Basic level (degree of Bachelor), Högskolan i Gävle, Akademin för hälsa och arbetsliv, Avdelningen för socialt arbete och psykologi; 2014.

[75] Eriksson M (2017). The Swedish communicative development inventory III Parent reports on language in preschool children. Int J Behav Dev.

[76] Bernier A, Carlson SM, Whipple N (2010). From external regulation to self-regulation: early parenting precursors of young children’s executive functioning. Child Dev.

[77] Gormley WT, Phillips D, Newmark K, Welti K, Adelstein S (2011). Social-emotional effects of early childhood education programs in Tulsa. Child Dev.

[78] Stowe RM, Arnold DH, Ortiz C (2000). Gender differences in the relationship of language development to disruptive behaviour and peer relationships in pre-schoolers. J Appl Dev Psychol.

[79] Seidman LJ, Biederman J, Faraone SV, Weber W, Mennin D, Jones J (1997). A pilot study of neuropsychological function in girls with ADHD. J Am Acad Child Adolesc Psychiatry.

[80] Brown RT, Madan-Swain A, Baldwin K (1991). Gender differences in a clinic-referred sample of attention-deficit disordered children. Child Psychiatry Hum Dev.

[81] Gordon M, Mettelman BB (1994). Gender differences in ADHD referrals: IQ, laboratory measures, and behavior ratings. ADHD/hyperactivity. Newsletter.

[82] Yeo M, Hughes E (2011). Eating disorders: early identification in general practice. Aust Fam Physician.

[83] Burger K (2010). How does early childhood care and education affect cognitive development? An international review of the effects of early interventions for children from different social backgrounds. Early Childhood Res Quarterly.

[84] Loeb S, Bridges M, Bassok D, Fuller B, Rumberger RW. How much is too much? The influence of preschool centers on children's social and. Cogn Dev. 2007. https://doi-org.ezp.sub.su.se/10.1016/j.econedurev.2005.11.005.

[85] Poarch GJ, Van Hell JG (2012). Executive functions and inhibitory control in multilingual children: evidence from second-language learners, bilinguals, and trilinguals. J Exp Child Psychol.

[86] Yang S, Lust, B. Testing effects of bilingualism on executive attention: comparison of cognitive performance on two non-verbal tests. BUCLD 29. In: Brugos A, Clark-Cotton MR, Ha S, editors. Online Proceedings Supplement. 2005. http://www.bu.edu/bucld/files/2011/05/29-YangBUCLD2004.pdf.

[87] Yang S, Lust B. Cross-linguistic differences in cognitive effects due to bilingualism: Experimental study of lexicon and executive attention in 2 typologically distinct language groups. In Caunt-Nulton H, Kulatilake S, Woo I-H. editors. BUCLD 31: Proceedings of the 31st annual Boston University Conference on Language Development Vol. 2. Somerville, MA: Cascadilla; 2007. p. 602–703.

[88] Costa A, Miozzo M, Caramazza A (1999). Lexical selection in bilinguals: do words in the bilingual’s two lexicons compete for selection?. J Mem Lang.

[89] Hermans D, Ormel E, Van Besselaar R, Van Hell JG (2011). Lexical activation in bilinguals’ speech production is dynamic: how language ambiguous words can affect cross-language activation. Lang Cogn Process.

[90] Bialystok E, Craik FI, Klein R, Viswanathan M (2004). Bilingualism, aging, and cognitive control: evidence from the Simon task. Psychol Aging.

[91] Pearson BZ, Fernández SC, Oller DK (1993). Lexical Deveopment in bilingual infants and toddlers: comparison to monolingual norms. Lang Learn.

[92] Kapa LL, Colombo J (2013). Attentional control in early and later bilingual children. Cogn Dev.

[93] McKean C, Mensah FK, Eadie P, Bavin EL, Bretherton L, Cini E, Reilly S (2015). Levers for language growth: characteristics and predictors of language trajectories between 4 and 7 years. PLoS One.

[94] Kalnak N, Peyrard-Janvid M, Forssberg H, Sahlén B. Nonword repetition – a clinical marker for specific language impairment in Swedish associated with parents’ language-related problems. PLoS One. 2014; 10.1371/journal.pone.0089544. 10.1371/journal.pone.0089544 PMC3933563 24586859

[95] Goodman R (1997). The strengths and difficulties questionnaire: a research note. J Child Psychol Psychiatry Allied Disciplines.

[96] Goodman R (2001). Psychometric properties of the strengths and difficulties questionnaire. J Am Acad Child Adolesc Psychiatry.

[97] Goodman R, Ford T, Simmons H, Gatward R, Meltzer H (2000). Using the strengths and difficulties questionnaire (SDQ) to screen for child psychiatric disorders in a community sample. Br J Psychiatry.

[98] Smedje H, Broman JE, Hetta J, von Knorring AL (1999). Psychometric properties of a Swedish version of the "strengths and difficulties questionnaire". Eur Child Adolesc Psychiatry.

[99] Malmberg M, Rydell AM, Smedje H (2003). Validity of the Swedish version of the strengths and difficulties questionnaire (SDQ-Swe). Nordic Journal of Psychiatry.

[100] Gelman A, Hill J (2006). Data analysis using regression and multilevel/hierarchical models.

[101] Preacher KJ, Hayes AF (2004). SPSS and SAS procedures for estimating indirect effects in simple mediation models. Behavior Research Methods, Instruments, and Computers.

[102] Sobel ME. Asymptotic confidence intervals for indirect effects in structural equation models. In: Leinhart S. editor. Sociol Methodol San Francisco: Jossey-Bass; 1982. p. 290–312.

[103] Freedman LS, Schatzkin A (1992). Sample size for studying intermediate endpoints within intervention trials of observational studies. Am J Epidemiol.

[104] MacKinnon D, Lockwood C, Hoffman J, West S, Sheets V (2002). A comparison of methods to test mediation and other intervening variable effects. Psychol Methods.

[105] Levene H. Robust tests for equality of variances. In: Olkin I, Hotelling H, et al., editors. Contributions to probability and statistics: essays in honor of Harold Hotelling: Stanford University Press; 1960. p. 278–92.

[106] Brown M, Forsythe A (1974). Robust tests for the equality of variances. J Amer Statist Assoc.

[107] Benjamini Y, Hochberg Y. Controlling the false discovery rate: a practical and powerful approach to multiple testing. J R Stat Soc 1995; Series B.57;1;289–300.

