# Supplementary material for: A protocol for a three-arm cluster randomized controlled superiority trial investigating the effects of two pedagogical methodologies in Swedish preschool settings on language and communication, executive functions, auditive selective attention, socioemotional skills and early maths skills
Source: BMC Psychol. 2018 Jun 19;6:29. doi: 10.1186/s40359-018-0239-y (PMC6006789; doi:10.1186/s40359-018-0239-y)
Supplement: Supplementary file 1 — Letter to parents. (PDF 282 kb) [file 40359_2018_239_MOESM1_ESM.pdf]

Stockholms universitet

Letter of informed consent
2016-08-17

Hillevi Lenz Taguchi
Professor
Barn- och ungdomsvetenskapliga institutionen
Avd. för förskollärarutbildning och förskoleforskning

To Guardians to children at X preschools

Dear Guardians to children attending X preschools

In a few weeks we start an interdisciplinary research project in your preschools that involves all children that have turned 4 and all 5 year olds. This interdisciplinary project involves three disciplines: early childhood education, linguistics and psychology. We want to study the effects of two of the most common pedagogical strategies with children in this age-group: investigative learning-processes in smaller groups of children and individual learning by ways of a leaning-program on a digital tablet. We want to know what effects they have on children attention abilities, language-development, general learning and social skills. So far no evidence-based research has been performed in Sweden in order to know what benefits these pedagogical strategies might have. Knowledge from this study might help develop early childhood education practices in ways that might benefit every single child. In order to evaluate the effects of the pedagogical strategies and methods, we want to know how they affect the individual child in terms of the variables mentioned. This is why we will observe and video-film the pedagogical practices as well as perform a number of simple tests on each of the children before and after 6 weeks of intervention with the two methods.

How is this research organized and performed?

Making an evidence-based study and the role of the control-group

In order to make possible valid results and impartial evaluation, we want only to compare groups of children that can be considered equal in terms of background factors. Therefore the study will start by asking you as the guardian of the child, well as the staff, a few questions about each of the children. The different preschools will be assigned the role of either control group, digital learning-method or investigative learning method. The pedagogical work will continue as planned in the control-group, whereas staff in the other two preschools will have to attend an evening course in order to supervise the digital learning method or perform the investigative learning processes with groups of children.

Stockholms universitet

In order to compare the different pedagogical methods, all children turning 4 during the fall semester of 2016 as well as the 5 year olds – including the children in the control preschools – will be invited to do the tests before and after the intervention period. Only by comparing the results on the group level of the control-preschool with the other two schools will we be able to know what effects the two methods might have on these two groups of children.

Researchers in the preschool

Researchers will visit the preschools during a period of approximately 10 weeks. We will educate and supervise staff as well as observe the practices. During the two first and two last weeks of the 10 week period the children will perform a number of playful standardized tests.

What kind of tests will be performed?

By tests we mean both the questions that we will ask you and the staff to answer as well as the playful standardized tests that each of the children aged 4 and 5 will take, focusing on attention, language, learning and social skills. Again, the same tests will be performed both before and after the 6 week intervention period. The tests will be performed at each of the preschool by trained staff with experience from testing children. The tests will be performed in a separate room at the preschool and will be videotaped in order to guarantee that they are properly performed. The tests are composed in a fashion that will be playful and fun for the child. The child is, for example, asked to point at different images, listen to a story and tell the adult what the story was all about or asked to perform a task that requires attention skills. In order to know how the interventions are going and what is going on in the control group preschool, the researchers will visit occasionally to observe and make video films of every day practices.

Attention-tests on a smaller group of children

A smaller amount of the 4 and 5 year olds will be asked to take a specific attention test where we measure the flow of brainwaves only in the parts of the brain that become active when children attend to a task and try to dismiss other disturbances. The child is invited to sit in a comfortable chair and listen to a story while looking at images and wearing a hat that has a small number of electrodes in it. The child will not feel anything from the hat, but the electrodes will register the brain-waves at those particular places where we can determine whether or not the child is paying attention to the voice that reads the story, as well as to what extent the child manages to disregard the added disturbing sounds in the headphones. All the child does is trying to listen to the story in one of the ears of the headphone. The hat can be compared to a stethoscope used to listen to the heart but showing the flow of the brain-waves instead without the child noticing this at all. The researchers performing these tests come from a well-established lab at Stockholm University, where they mostly do this with smaller babies that

Stockholms universitet

come to the lab with their guardians in order to measure brain-waves of aspects of language-development. Children usually find this a fun and engaging activity. Since the procedure is costly, only a smaller amount of children will be asked to participate from all three groups. The guardians of the participating children will get more information ahead of the test. The lab will be set up in a quiet room at the preschool or a nearby preschool, to which staff who is close to the child will accompany the child.

In relation to all these tests, please remember that it is not the individual child that will be evaluated, but the pedagogical methods at the group level. The children's test results will only be analyzed at the group level. Individual data will be handled according to national research ethics guidelines (www.vr.se).

Additional information

Personal data including the below form

All research data will be stored in a secured safe at Stockholm University. The registration of personal data is done following the laws of *Offentlighets- och Sekretesslagen* as well as the law for registration of personal data *PUL 26 §* . There is a specifically appointed and responsible solicitor at Stockholm University who will answer to all questions and requests according to this law. The project-leader is, however, responsible for the data collection.

Digital data

All digital data and films will be sawed on external hard drives and encrypted. It will be stored according to criteria from the *Swedish National Data Inspection* according to the rules of personal data in a data based owned and controlled by Stockholm University. The researchers will also ask staff to be entrusted with some of the pedagogical documentation produced during the intervention period. These will also be digitalized and treated the same fashion as all other research data.

Access to data

Only researchers who are tied to the project will have access to data and be allowed to analyze the data.

Stockholms universitet

Presentation of research results

The results of the study will be presented at the project web-page and at information-meetings with staff and guardians. The results will only be presented at the group level. No information that can be traced to an individual child, group or preschool will be presented.

The results will also be presented in the form of international research articles, reports, scientific lectures and papers presented at international conferences. A popular science book will be written from the major project. No photographs or film-sequences where a child, staff or preschool can be identified will be used or published. Films and photographs will only be used in order to analyze or validate analyzes performed.

Voluntarily participation

Participation in the project is voluntary and you can decide to withdraw your child's participation at any time without explaining why. If you want to withdraw the participation of your child, please call or write to the project leader Hillevi Lenz Taguchi, who will see to that no more data collections will be made with your child.

Contact information

Professor Hillevi Lenz Taguchi

Barn och ungdomsvetenskapliga institutionen

Stockholms universitet

E-mail: hillevi.lenz-taguchi@buv.su.se,

Telefon: +46 8 12 07 63 82

Mobil: +46 73 46 12 533

Please keep this information at home and fill in the form below. We are grateful if you would return the form within the week ahead and give it to staff at the preschool or to any of the researchers.

Any questions can be answered by Hillevi Lenz Taguchi. I prefer that you e-mail first and I will call you up at the time it suits you.

The research project web-page

Information from the project will be published at the web-page: **www.buv.su.se/hjärnvägar**

Stockholms universitet

INFOMED CONSENT FORM

Hereby I declare that I have read the information about the project. I will mark one of the boxes in order to show my informed consent or that I decline to let the child to whom I am the guardian to participate in the project. The signature of both guardians if more than one is required. Please return only this form to staff at your child's preschool as soon as possible. Thank you!

Mark only one of the alternatives below:

□ Yes, I agree to let my/our child participate in the tests, video recordings and observations that will be produced during the project.

□ No, I/we decline participation in the project.

Date:___________________

Signature guardian 1:

____

Please print your name above

Address guaridan 1:____

E-mail guardian 1:____

Telephone guardian 1:____

Signature guardian 2: Date if different from above: _____________________________

____

Please print your name above

Address guardian 2:____

E-mail address guardian 2:____

Telephonenumber guardian 2:____

Name of the child:____

Birth year and month:_____________________________________(*not* the last 4 numbers)

Preschool/Groupname:____
